# Whole-exome sequencing of pancreatic cancer defines genetic diversity and therapeutic targets

**DOI:** 10.1038/ncomms7744

**Published:** 2015-04-09

**Authors:** Agnieszka K. Witkiewicz, Elizabeth A. McMillan, Uthra Balaji, GuemHee Baek, Wan-Chi Lin, John Mansour, Mehri Mollaee, Kay-Uwe Wagner, Prasad Koduru, Adam Yopp, Michael A. Choti, Charles J. Yeo, Peter McCue, Michael A. White, Erik S. Knudsen

**Affiliations:** 1 Simmons Cancer Center, UT Southwestern Medical Center, Dallas, Texas 75390, USA; 2 Department of Pathology, UT Southwestern Medical Center, Dallas, Texas 75390, USA; 3 Department of Cell Biology, UT Southwestern Medical Center, Dallas, Texas 75390, USA; 4 Eppley Institute for Research in Cancer and Allied Diseases, University of Nebraska Medical Center, Omaha Nebraska 68198, USA; 5 Department of Surgery, UT Southwestern Medical Center, Dallas, Texas 75390, USA; 6 Department of Pathology, Thomas Jefferson University Philadelphia Pennsylvania 19107, USA; 7 Department of Surgery, Thomas Jefferson University, Philadelphia Pennsylvania 19107, USA

## Abstract

Pancreatic ductal adenocarcinoma (PDA) has a dismal prognosis and insights into both disease etiology and targeted intervention are needed. A total of 109 micro-dissected PDA cases were subjected to whole-exome sequencing. Microdissection enriches tumour cellularity and enhances mutation calling. Here we show that environmental stress and alterations in DNA repair genes associate with distinct mutation spectra. Copy number alterations target multiple tumour suppressive/oncogenic loci; however, amplification of *MYC* is uniquely associated with poor outcome and adenosquamous subtype. We identify multiple novel mutated genes in PDA, with select genes harbouring prognostic significance. *RBM10* mutations associate with longer survival in spite of histological features of aggressive disease. *KRAS* mutations are observed in >90% of cases, but codon Q61 alleles are selectively associated with improved survival. Oncogenic *BRAF* mutations are mutually exclusive with *KRAS* and define sensitivity to vemurafenib in PDA models. High-frequency alterations in Wnt signalling, chromatin remodelling, Hedgehog signalling, DNA repair and cell cycle processes are observed. Together, these data delineate new genetic diversity of PDA and provide insights into prognostic determinants and therapeutic targets.

## Abstract (web summary)

Diagnosis of pancreatic ductal adenocarcinoma (PDA) has poor long-term survival rates with limited therapy options. Here Witkiewicz *et al.* use microdissection and whole-exome sequencing to identify novel recurrent PDA mutations, highlighting the genetic diversity of this aggressive cancer.

## Graphical abstract

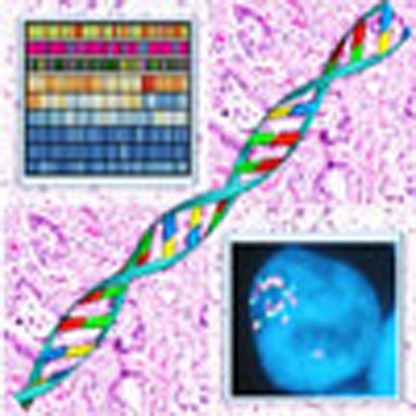

Pancreatic ductal adenocarcinoma (PDA) portends a poor prognosis, with a 5-year survival rate of ∼6% (refs 1, 2, 3). This poor outcome is related to multiple factors, including the relatively late stage of diagnosis, many patients presenting with unresectable disease, and therapy recalcitrance resulting in disease recurrence in spite of operable disease and systemic therapy^1^. Genetic analysis of PDA yielded insights related to altered signalling pathways^4^,^5^; however, unlike other cancers the number of sequenced PDA genomes has been relatively modest. This reflects the difficulty of sequencing a tumour that is dominated by presence of stromal and inflammatory cells^2^,^6^. Tumour cellularity in samples subjected to whole-exome or -genome sequencing represents a known barrier in obtaining high quality data; therefore, many studies recommended >60% tumour cell content in the samples^7^. To facilitate mutation detection and remove contaminating non-neoplastic tissue the initial PDA sequencing effort employed patient-derived xenografts and cell lines^5^. This approach resulted in detecting an average of 48 somatic mutations per model sequenced. Subsequent work employed exome sequencing on 99 clinical cases of PDA, and applied the q-pure algorithm to overcome contamination with non-neoplastic tissue, yielding an average of 26 mutations per case^4^.

Here 109 micro-dissected PDA cases annotated with outcome data were subjected to whole-exome sequencing. The data from this analyses demonstrates that there is substantial genetic diversity within PDA with a mutation frequency comparable to other solid tumours. Alterations in mutation spectra and burden are associated with hallmark pancreatic cancer risk factors. Increased copy number variation indicative of chromosomal instability is associated with poor outcome. Amplification of the locus involving the *MYC* oncogene are specifically associated with poor outcome and association with adenosquamous subtype of PDA. In contrast, select mutated genes (for example, *RBM10*) are associated with longer patient survival. Diversity within the canonical KRAS signalling pathway defined prognostic features of the disease and the potential for targeted therapy of BRAF-mutated cases. The mutation and copy number alterations frequently involved important signalling pathways that contribute to tumour development and represent therapeutic targets.

## Results

### Tissue cell enrichment and mutation burden

**Figure 1:**
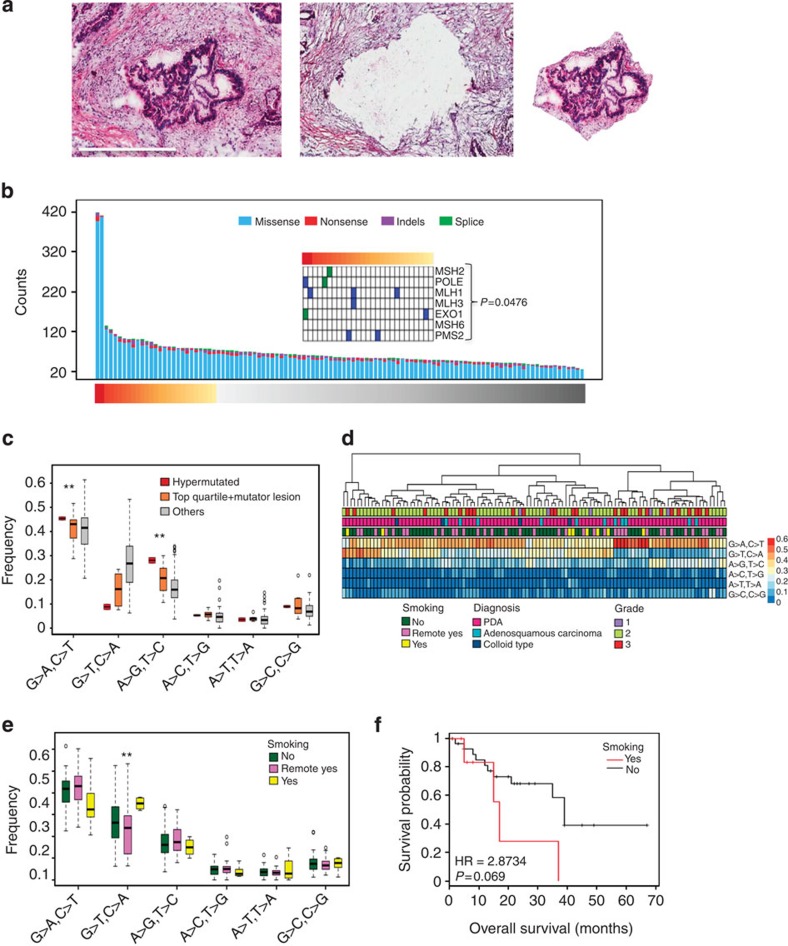
Overall mutational landscape of PDA and association with etiological features of disease. (**a**) Representative image of the needle dissection approach used to enrich tumour cells for whole-exome sequencing (scale bar, 600 μM) (**b**) Mutational burden across the sequenced cohort. The presence of genetic alterations in genes associated with mutator phenotypes are shown for the cases with the top 25% of mutational burden. Association with mutational burden was determined by a hypergeometric test. (**c**) Mutation spectra of the hypermutated cases, top quartile cases with mutator mutations and others (** denotes *P*<0.05, Student's *t*-test). The boxes show the distance between the first and third quartile with the whiskers extending up to 1.5 times the interquartile range. (**d**) Unsupervised hierarchical clustering of cases based on mutation spectra. (**e**) Mutation spectra based on smoking status, the increase in G>T transversion is significant (*P*<0.05, Student's *t*-test). The boxes show the distance between the first and third quartile with the whiskers extending up to 1.5 times the interquartile range. The unfilled circles represent possible outliers. (**f**) Association of PDA smoking status with overall survival. Hazard ratio and *P* value were obtained from Cox proportional hazard test.

To enhance tumour cellularity in PDA patient samples, needle dissection was employed to significantly enrich PDA tumour cells from the surrounding microenvironment (Fig. 1a). This approach was applied to a total of 109 surgically resected PDA cases annotated with clinical outcome and etiological features (Table 1). The cohort had a median survival of 21 months that is consistent with historical survival of resected PDA^8^,^9^. Of standard pathological features, grade 3 was associated with poor outcome, while nodal status and adenosquamous subtype trended towards poor outcome (Supplementary Figs 1 and 2). Enriched tumour cells were utilized as the substrate for whole-exome sequencing (Methods). Average tumour purity exceeded 50% and the average sequencing depth was ∼50 × . In prior studies sequencing PDA^4^, 26 mutations were detected per case representing a relatively low mutational burden when compared with the majority of solid tumours (Supplementary Figs 3 and 4). The average mutation burden observed in our cohort was 67 non-synonymous events per case, equivalent to multiple other solid-tumour types (Fig. 1b, Supplementary Data 1, Supplementary Figs 3–5). Sanger sequencing was used to validate 248 genetic events with validation occurring in 92% of cases (Supplementary Data 1). In addition, p53 immunohistochemistry performed across the cohort demonstrated the expected strong correlation between *TP53* mutation and stabilization of the protein (Supplementary Fig. 6). To determine whether deeper sequencing would further increase the number of mutations detected, 21 cases were sequenced to ∼120 × depth. The majority of events identified with deeper sequencing had low allelic frequency or coverage depth, and only a small fraction of statistically significant mutations were identified (Supplementary Fig. 7 and Methods).

### Determinants of mutation burden and spectrum

Consistent with observations in other solid malignancies^10^,^11^,^12^, cases with the highest relative mutational burden harboured genetic lesions in mismatch repair genes, known to be associated with a mutator phenotype (Fig. 1c). These cases also displayed a mutation spectrum consistent with mismatch repair deficiency, T>C transition at CTG tri-nucleotides^11^,^13^ (Fig. 1c and Supplementary Fig. 8). The overarching mutational spectra of the PDA cohort were dominated by C>T mutations associated with age, and had minimal evidence for enriched APOBEC-associated mutagenesis at TCW tri-nucleotides (Supplementary Fig. 8). By hierarchical clustering it was apparent that the variable mutational spectra of individual PDA cases specified discrete subtypes (Fig. 1d). Smoking status was associated with an enrichment of C>A transversions that resembled the mutational ‘smoking signature' observed in other cancers for which smoking is a significant risk factor (Fig. 1e, Supplementary Figs 9–11)^12^,^14^. Smokers had generally poor outcome, with a median survival half that of non-smokers (Fig. 1f). Thus, etiological events associated with the development of PDA were identifiably associated with the mutational spectrum of the tumours.

### Copy number variation and the role of MYC

**Figure 2:**
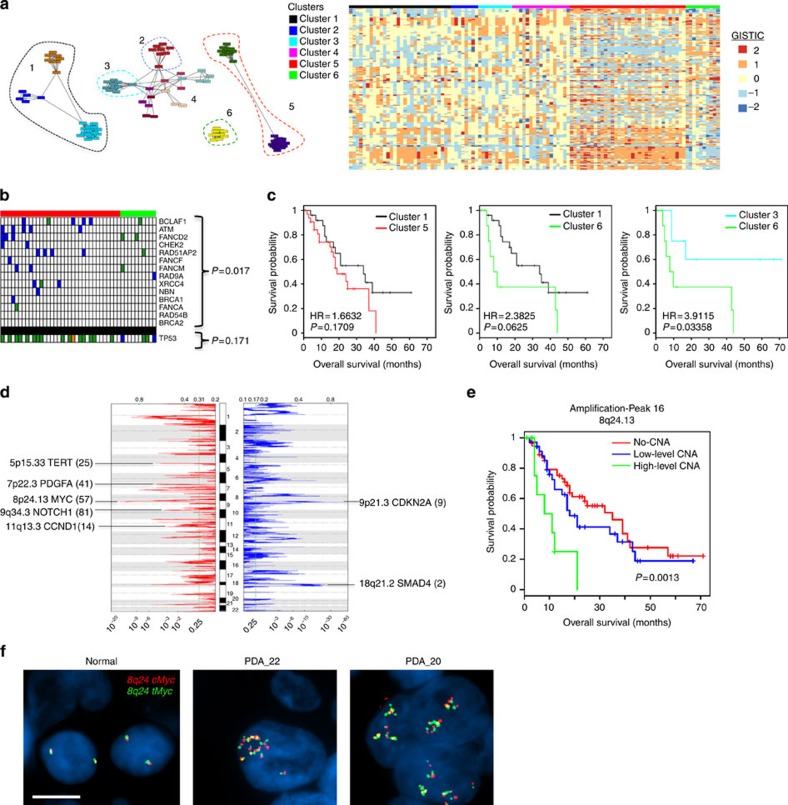
Copy number alterations in PDA. (**a**) APC clustering of PDA cases by CNV revealed several distinct clades and associated clusters. Clusters 5 and 6 exhibit higher overall CNV relative to the other clusters. (**b**) Clusters 5 and 6 are enriched for mutations or homozygous deletion of genes involved in double-strand break repair, but not in TP53. Significance was determined by hypergeometric test. (**c**) Cluster 5 and 6 trend towards poor survival relative to clusters with less CNV. Hazard ratio and *P* values were obtained from Cox proportional hazard test. (**d**) GISTIC analysis of cases reveals chromosomal regions that are significantly deleted/amplified. (**e**) Kaplan–Meier analysis of the association of the 8q24.13 locus amplification with overall survival. *P* value was obtained from Cox proportional hazard test. (**f**) Fluorescence *in situ* hybridization with MYC break apart probes demonstrates amplification in the absence of translocation at the MYC locus (Scale bar, 5 μM).

Affinity propagation clustering (APC) was used to delineate deterministic patterns of commonality associated with copy number alterations (Fig. 2a, left panel) and partitioned cases in discrete subtypes with increasing genetic complexity (Fig. 2a, right panel). Cases with high levels of amplifications/deletions (clusters 5 and 6) were significantly over-represented for alterations of genes involved in DNA break repair, but not *TP53* (Fig. 2b). Similar results were observed with standard hierarchical clustering methods based on Euclidean distance (Supplementary Figs 12 and 13). Both cluster 5 and 6 harboured poor outcome relative to other clusters with fewer copy number changes (Fig. 2c, Supplementary Figs 12 and 13). GISTIC analysis defined significant common regions of amplification and deletion that harbour multiple oncogenes (for example, *MYC* and *CCND1*) and tumour suppressors (for example, *SMAD4* and *CDKN2A*) (Fig. 2d and Supplementary Data 2). Interrogation of amplified and deleted regions for association with survival (Supplementary Data 2) revealed that the amplification of the 8q24 locus, harbouring the *MYC* oncogene, was uniquely associated with poor outcome (Fig. 2e, Supplementary Data 2). While *MYC* overexpression has been shown to facilitate the development of pancreatic cancer in mouse models^15^, little analysis has been performed in patient specimens. The amplification of *MYC* was confirmed by fluorescent *in situ* hybridization (Fig. 2f). The *MYC* amplified cases did not have a higher mutation burden or association with other hallmark mutations of PDA; however, amplification was significantly over-represented in the adenosquamous subtype of pancreatic carcinoma (Supplementary Fig. 14). Evaluation of precursor lesions pancreatic intraepithelial neoplasia (PanIN) associated with invasive disease also revealed *MYC* amplification (Supplementary Fig. 15). These data suggest a role of *MYC* in initiation and progression of this exceedingly aggressive form of PDA. Consistent with this observation, in a *MYC*-driven mouse model of pancreatic cancer, the tumours exhibited adenosquamous histology and stained positively for p63, an established marker of squamous differentiation (Supplementary Fig. 16).

### Significantly mutated genes in pancreatic cancer

**Figure 3:**
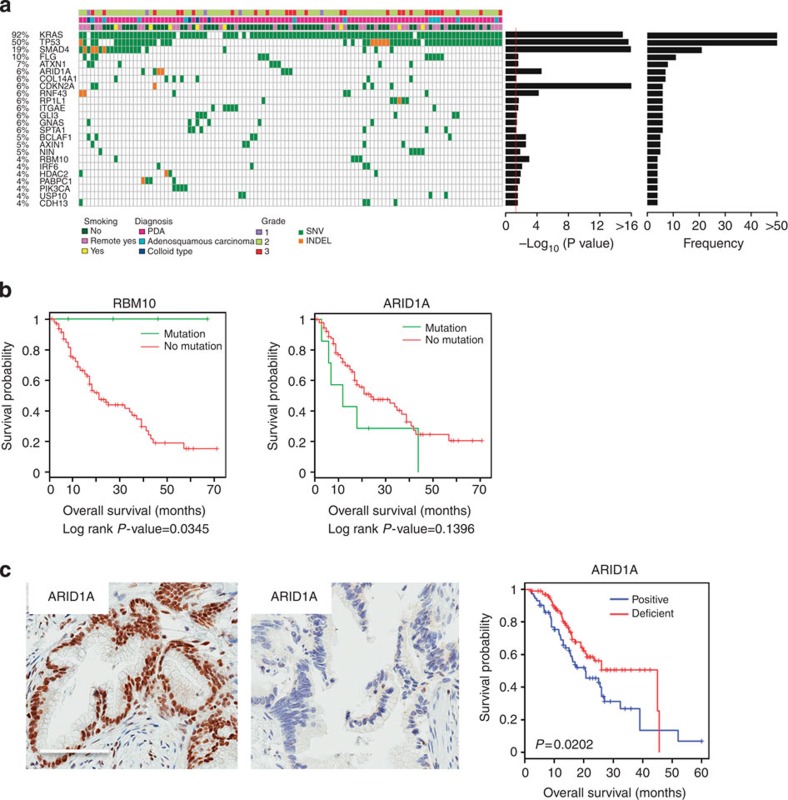
Significantly mutated genes in PDA. (**a**) Mutational significance was determined for SNV and INDELS from the 109 sequenced cases using MutsigCV. Genes were subjected to unsupervised clustering, and the frequency and *P* value as determined by MutsigCV are shown. (**b**) Kaplan–Meier analysis of select significantly mutated genes. *P* value was obtained from Cox proportional hazard test. (**c**) IHC was used to confirm loss of ARID1A protein in a large cohort of PDA cases (scale bar, 150 μM). Diminished ARID1A protein level was associated with overall survival. *P* value was obtained from Cox proportional hazard test.

MutSigCV analysis^16^ of 109 PDA/normal tissue pairs revealed 24 significantly mutated genes occurring in >3.5% of cases (Fig. 3a, Supplementary Data 3). For comparison. similar analysis performed using data from prior sequencing effort^4^ revealed only four non-synonymous mutations (Supplementary Figs 17–18). The identification of *KRAS, TP53, CDKN2A* and *SMAD4* confirmed that our approach detected known alterations promoting PDA tumorigenesis. *GNAS* mutations were present in all four colloid carcinomas and two conventional PDAs. Most *GNAS* mutations were in hotspot codon 201 (R201C and R201H) (Supplementary Figs 19 and 20). Consistent with recent studies^17^, all *GNAS*-mutated invasive carcinoma cases were derived from a common precursor, intraductal pancreatic neoplasm (IPMN). Four of the six PDAs harbouring *GNAS* alteration had concomitant mutations in *KRAS* (Supplementary Data 1). Two other genes reported as mutated in IPMN, *RNF43 *and *RBM10*, were detected in six and four conventional PDA cases, respectively. Notably, PDAs harbouring *RNF43* and *RBM10* alterations did not arise from IPMN and there was no association between these genes and *GNAS* mutations in this cohort. We also identified a number of ‘cancer genes'^18^ as significantly mutated in PDA, including *BCLAF1* (5% of cases), *IRF6* (4% of cases), *FLG* (10% of cases), *AXIN1* (5% of cases), *GLI3* (6% of cases) and *PIK3CA* (4% of cases).

### Mutated genes associated with pancreatic cancer prognosis

Selected mutations were associated with pathological features of disease or survival (Fig. 3b and Supplementary Table 1). *RBM10* is critical regulator of alternative splicing that was recently identified as significantly mutated in lung cancer^19^,^20^. Remarkably, *RBM10* mutations were associated with enhanced survival (Fig. 3b), although all *RBM10-*mutated cases were of high grade, pT3 stage and harboured lymph node metastasis in three of four cases (Supplementary Fig. 21). Mutations in the chromatin remodelling gene ARID1A trended towards poor survival (Fig. 3b). Immunohistochemical analyses of additional 296 PDA cases demonstrated that ARID1A protein deficiency was significantly associated with poor outcome in this expanded cohort (Fig. 3c, Supplementary Table 2). The loss of ARID1A protein is similarly observed in multiple PDA cell lines, although whether this event is functionally significant is unknown (Supplementary Fig. 22). Loss of ARID1A is known to induce vulnerability to ARID1B depletion in other cancer models^21^ and we could confirm this finding in ARID1A-deficient PDA cell lines, indicating that loss of protein has a functional consequence (Supplementary Fig. 22). To further explore gene interactions, deterministic clustering was performed followed by analysis of exemplar cases (Supplementary Fig. 23). This work further reinforced the significance of genetic alterations beyond *KRAS* and *TP53*, as coordinate mutation of *ARID1A* delineated a cluster with worse prognosis (Supplementary Fig. 23). Correlation of mutations with clinicopathologic data revealed specific somatic aberrations associated with PDA grade, presence of lymph node metastasis and adenosquamous histology (Supplementary Table 1).

To comprehensively interrogate significant mutations in PDA clinical cases, MutSigCV analysis was performed using combined data from current and 99 previously reported PDA cases^4^. The combined 208 cases yielded no additional high-frequency mutated genes, however, multiple lower frequency (<2.5% recurrence) cancer-related genes were identified including *ATM, ARID2, TGFBR2* and *ACVR1B* (Supplementary Fig. 24). This additional discovery is consistent with the added power of combined data analysis being required for a full accounting of cancer genomes^18^.

### Genetic diversity in the KRAS pathway

**Figure 4:**
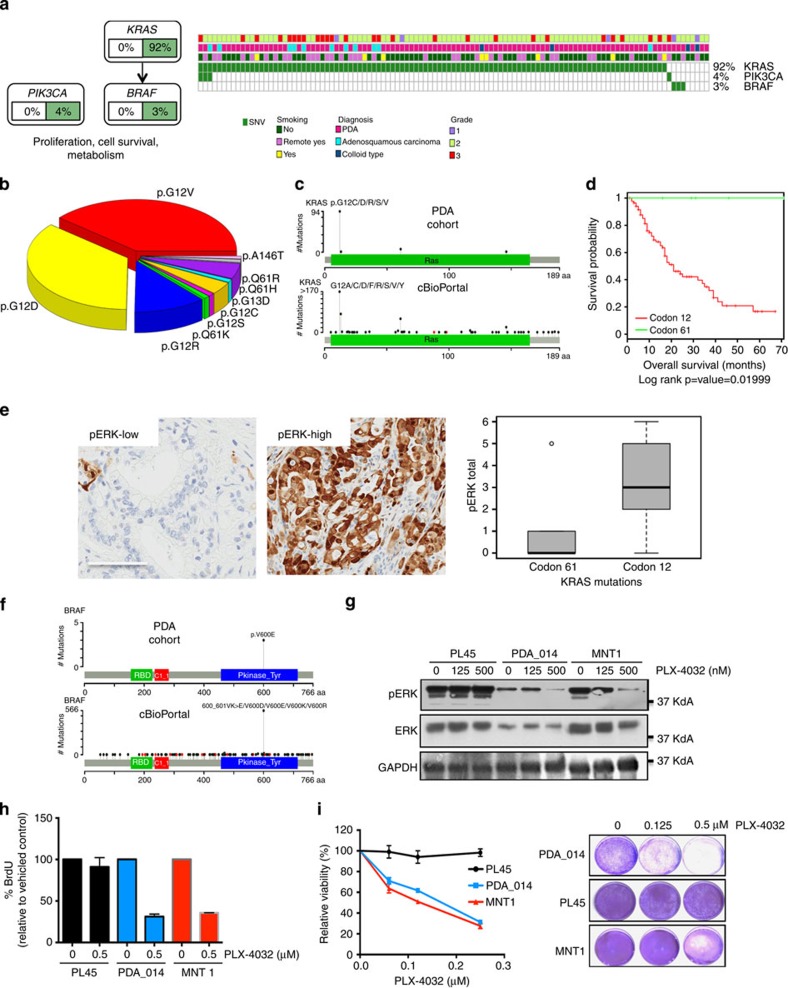
KRAS/BRAF-Pathway in PDA. (**a**) Oncomap showing the distribution of KRAS, BRAF and PIK3CA mutation in the cohort. (**b**) KRAS mutations in the cohort exhibit a diverse spectrum concentrated at known oncogenic codons 12, 13 and 61 (**c**) Analysis of KRAS mutations in the PDA cohort in comparison with all cancer cases in cbioportal (**d**) Kaplan–Meier analysis of codon 12 versus codon 61 mutated cases. Kaplan–Meier analysis of codon 12 versus codon 61 mutated cases. *P* value was obtained from Cox proportional hazard test. (**e**) Representative staining of pERK in pancreatic cancer cases (scale bar, 150 μM), and the association of pERK staining in PDA cases with KRAS codon 12 versus codon 61 mutations. The boxes show the distance between the first and third quartile with the whiskers extending up to 1.5 times the interquartile range. (**f**) Analysis of BRAF mutations identified in the PDA cohort in comparison with the analysis of all cancer cases in bioportal. (**g**) Impact of PLX-4032 on the phosphorylation of ERK in the indicated cell lines. PL45 is KRAS mutated, PDA_014 is a patient-derived cell line harbouring BRAF V600E and MNT1 is a BRAF V600E melanoma cell line (**h**) Impact of PLX-4032 on cell cycle progression in the indicated cell lines (data analysis was performed in triplicate and error bars denote s.d.). (**i**) Impact of PLX-4032 on viability in the indicated cell lines (data points are from at least six values and error bars denote s.d.).

PDA is dominated by mutations in *KRAS* that were detected in 92% of the cases in our cohort (Fig. 4a). The majority of KRAS mutations occurred in codon 12, although mutations also occurred in codons 13 and 61 (Fig. 4b and c), consistent with other sequencing studies^4^,^5^ (Fig. 4c). It has been suggested that different *KRAS* mutations harbour diverse biological activity^22^. Notably, while all mutations affecting codon 12 exhibited similar association with survival (Supplementary Fig. 25), cases mutated at codon 61 had a remarkably favourable prognosis in this cohort (Fig. 4d). Interestingly, as we observed in the *RBM10* mutant tumours, cases harbouring mutations at codon 61 paradoxically exhibited histological and clinical features indicative of poor survival (Supplementary Fig. 26). When assessed for pERK staining, cases with *KRAS* mutations at codon 61 exhibited less ERK activation as compared with cases with other *KRAS* alleles (Fig. 4e). This finding suggests that in PDA, *KRAS* mutant allele status potentially determines the quality of RAS pathway activation and the prognosis of disease.

While prior sequencing studies have shown that a small subset of PDA cases harbour wild-type *KRAS*, the genomic driver(s) of these tumours has remained obscure. Here we found that *KRAS* wild-type PDA exhibit somatic lesions in known oncogenes that are also RAS effector proteins (Fig. 4a and Supplementary Figs 27–30). Importantly, the *PIK3CA* mutations and *BRAF* mutations, occurring in cases with wild-type KRAS, are known to be mechanistically activating and are enriched in other tumour types (Fig. 4f and Supplementary Figs 27–30). In cases with wild-type *KRAS, BRAF* and *PIK3CA* a number of cancer-associated genes representing potential drivers were identified (for example, *STK11, GNAS, CHEK2* and *RB1*) (Supplementary Fig. 28). *BRAF* V600E mutations, occurring at a frequency of 3%, were mutually exclusive with *KRAS* mutations (Supplementary Fig. 29). While the expression of *BRAF* V600E would suggest that it is a driver event based on mouse models^23^, no endogenous model of this form of pancreatic cancer had been developed. One of the *BRAF* V600E cases (PDA_014) was utilized to develop a cell line that maintained the *BRAF* mutation (Supplementary Fig. 30). *BRAF* V600E PDA cell line was equally sensitive as MNT1 melanoma cells to the FDA-approved BRAF inhibitor PLX-4032, while KRAS driven cells (PL45) are resistant (Fig. 4g-i). Together, these data suggest that genetic analysis of PDA could identify a subset of patients, *albeit* small, who may benefit from targeted therapy along the KRAS/BRAF axis.

### Frequently mutated pathways in pancreatic cancer

**Figure 5:**
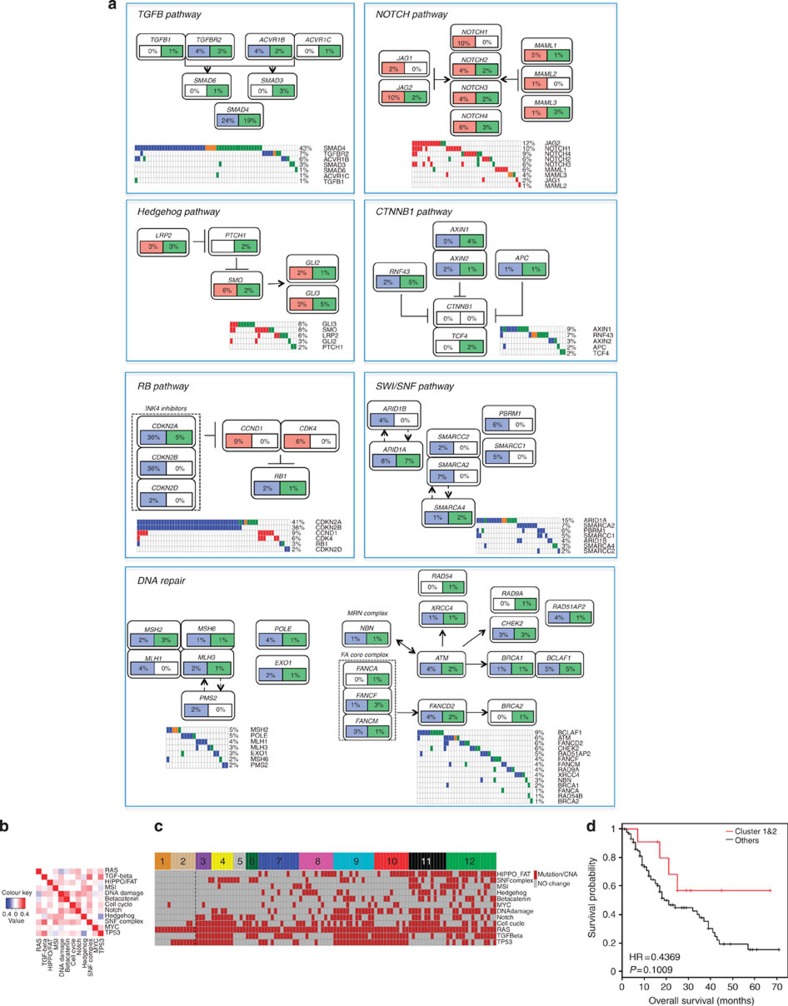
Pathways in PDA. (**a**) Oncomaps and schematics of pathways with combined genetic lesions occurring at a frequency >15% in the PDA cohort. (**b**) Correlation between pathways in the PDA cohort reveals relatively weak interactions between pathways. (**c**) Random forest-based clustering of pathways. (**d**) Overall survival related to genetic alterations targeting KRAS and TP53 in clusters 1 and 2 versus all others. *P* value was obtained from Cox proportional hazard test.

In addition to heterogeneity within the core KRAS pathway, we detected multiple additional pathways that were genetically altered at high frequency (>20%) in PDA (Fig. 5a, Supplementary Figs 31–33). Specifically, we found that the TGF-ß pathway is disrupted via a combination of largely mutually exclusive events in addition to the frequent disruption of *SMAD4*. This includes loss of *TGFBR2/TGFBR1*, as well as mutations in *ACVR1B*, which is a newly identified cancer-associated gene in the pathway^18^. Similarly, in concert with frequent deletion of *CDKN2A* and *CDKN2B, CDK4* and *CCND1* amplification and *RB1* loss was observed in the RB pathway. Among signalling pathways, beta-catenin and NOTCH pathways exhibited frequent alterations indicative of oncogenic activation. Multiple Fanconi Anaemia genes as well as *ATM, CHEK2, BCLAF1, BRCA1* and *BRCA2* were observed to be mutated or deleted at relatively high frequency with alterations targeting some facet of DNA repair occurring in >35% of cases. Genetic lesions in chromatin remodelling SWI/SNF pathway occurred in 42% of the cases. In addition to these well-described pathways, we observed frequent aberrations that impinge on histone modification and the FAT/HIPPO pathway (Supplementary Figs 31–32). Each pathway was interrogated for association with outcome (Supplementary Fig. 34). Only DNA repair-associated pathways had a trend towards poor outcome, while beta-catenin signalling trended towards improved outcome. In general, the associations between pathways were limited as determined by Pearson's correlation (Fig. 5b). Random Forest and APC clustering were used to define subtypes of PDA based on altered pathways, and demonstrated marked diversity of combinations of deregulated pathways (Fig. 5c and Supplementary Fig. 35). While tumours with isolated KRAS-pathway alterations alone or in combination with TP53 have a poor prognosis, tumours with more complex pathway deregulations trended towards even poorer outcome (Fig. 5d). Many of the highlighted pathways represent therapeutic targets that are actionable in preclinical models and, in some cases, in the clinic (Table 2). For example, BRAF V600E has been shown to be a potential target in melanoma and other cancers. Loss of *RNF43* or *AXIN1* are associated with sensitivity to porcupine and tankyrase inhibitors targeting the beta-catenin pathway^24^, while deletion of *CDKN2A* or amplification of *CDK4/CCND1* confer sensitivity to CDK4/6 inhibitors^25^. Deficits in Fanconi Anaemia genes can be targeted by cross-linking agents^26^, a finding readily observed in cell lines harbouring homozygous deletion of *FANCF* (Supplementary Fig. 36). Similarly, loss of BRCA function is associated with response to PARP inhibitors^27^. A number of these genetic alterations remain actionable in the presence of an activating KRAS mutation and could provide the opportunity for genetically targeted therapy in PDA.

## Discussion

In total, the data herein demonstrates that PDA is a genetically diverse disease harbouring mutational burden similar to other solid malignancies. Due to the enrichment approach utilized, we identified multiple significantly mutated genes that have not been previously described in PDA and provide genetic insights into the etiology, prognostic features and potential therapeutic targets.

Risk factors for PDA, include smoking and deficits in DNA mismatch repair as exemplified by Lynch Syndrome^28^,^29^,^30^. We observed that smoking status in PDA was associated with a mutation spectra similar to other smoking-associated tumours^13^,^14^. We also found that select cases exhibit a ‘mutator' phenotype, suggesting a contribution of mismatch repair deficiency in tumorigenesis^12^.

In the analysis of copy number, we identified many regions that have been previously identified by CGH and other methods^31^. In general, tumors with more copy number alterations (indicative of chromosomal instability) exhibited mutations in DNA break repair genes and trended toward poor prognosis. Importantly, we discovered that *MYC* amplification was associated with poor prognosis and adenosquamous histology. We did not identify mutations in *UPF1,* which has been recently reported to be selectively mutated with high frequency in pancreatic adenosquamous carcinoma^32^. In spite of a comprehensive unbiased survey of amplicons/deleted regions only one locus (MYC) was associated with outcome; however, clearly there is variation in the overall magnitude of copy number alterations, suggesting that there are perhaps distinct drivers for the chromosomal instability observed in PDA.

Using MutSigCV and pathway analysis, multiple genes and pathways altered at high frequency were identified, a subset of which associated with survival. *ARID1A* was confirmed as a marker of poorer outcome using an independent cohort, and *RBM10* mutation was associated with longer survival. We also uncovered a number of novel *albeit* rare alterations in signalling pathways important in PDA biology. While *CDKN2A* loss is common in PDA and contributes to the aberrant cell cycle progression, loss of *RB1* or *CCND1* amplification has not been previously reported. In PDA tumours harbouring wild-type KRAS, we identified *BRAF* and *PIK3CA* mutations at oncogenic hotspots expanding the spectrum of oncogenic drivers in PDA. Importantly, a cell line developed from BRAF V600E-mutated PDA was equally sensitive as melanoma cells to FDA-approved BRAF inhibitor PLX-4032. In addition to these rare, potentially, clinically actionable events, several pathways that were genetically altered at high frequency (>20%) in our study could yield selective therapeutic sensitivities. Genetic lesions targeting some facet of DNA repair or chromatin remodelling SWI/SNF pathway occurred in >35% and 42% of PDA cases, respectively. Alteration in WNT/beta-catenin pathway were common and included loss of RNF43 or AXIN1 conferring sensitivity to porcupine and tankyrase inhibitors targeting. A number of these genetic alterations remain actionable in the presence of an activating KRAS mutation and could provide the opportunity for genetically targeted therapy in PDA.

Together this expanded genetic framework of PDA reveals disease complexity that will contribute to future efforts of disease modelling and patient stratification for treatment.

## Methods

### Tissue collection and microdissection

All tissues were collected under approved protocols of the Thomas Jefferson University Institutional Review Board and UT Southwestern Medical Center Institutional Review Board that require the informed consent of all patients. Cases represented diverse histological subtypes including colloid (*n*=4), adenosquamous (*n*=11) and ductal carcinoma, NOS (*n*=94). The majority of the analysed tumours were stage I (*n*=5) or II (*n*= 97); the remainder were stage III (*n*=6) or IV (*n*=1). For stage IV case tissue form the primary pancreatic tumour was analysed. About 7-μM thick slides were cut from frozen tissue and lightly stained with haematoxylin. Tumour epithelial cells were dissected manually using a 14 gauge needle under a dissecting microscope (Leica Microsystems). DNA was extracted using the QIAamp DNA Mini Kit. Between 500 ng and 1.5 μg of enriched tumour DNA was recovered from each case. Analysis of KRAS mutation and other tumour-associated mutations demonstrated that average tumour cellularity >50%. Germline DNA was obtained from normal tissue resected at surgery (*n*=105) or peripheral blood (*n*=4).

### DNA quality control and sequencing

Recovered DNA was quantified by Nanodrop and Qubit using standard procedures. Integrity of genomic DNA was confirmed by gel electrophoresis. A total of 109 tumour/normal pairs passed the quality control to move forward for whole-exome sequencing. TruSeq Exome Enrichment (FC-121-1048) and Nextera Exome Enrichment (FC-140-1003) kits were utilized to build the sequencing libraries as per manufacturer's protocol. The DNA was then subjected to paired-end whole-exome sequencing using Illumina HiSeq2500 instruments. The sequence reads were aligned to the human genome (Build-UCSC hg19) using the BWA alignment algorithm^33^. A general sequencing depth of 51.28 × (95% confidence interval (CI): 49.5 and 53.1) was observed for sequenced cases, and represented 99.26% (95% CI: 99.20–99.31%) of the target sequences. Across the target sequences, 91.51% (95% CI: 90.97–92.05%) had >15 read coverage, and on average 2.178 × 10^9^ bases were sequenced per case. For 21 cases, the sequencing depth was doubled with an average number of 5.523 × 10^9^ bases sequenced per case. The average fold coverage was 123.01 (95% CI: 115.4−130.6). Across target sequences, 94.74% (95% CI: 93.41–96.07) had >15 read coverage. With the deeper sequencing relatively few additional mutation were discovered. The increased depth largely served to detect mutations with low sequence coverage and allele frequency. These data are summarized in Supplementary Fig. 7.

### Informatics pipeline

Somatic point mutations and INDELS in the tumour tissues compared with their corresponding normal tissues were identified using the MuTect^34^ and VarScan2 algorithms^35^, respectively, with default parameter settings. The mutations were annotated using Broad Institute's Oncotator programme. Minimum of 14 reads covering a site in the tumour and 8 in the normal were required for mutation calling. Only INDELS with reference allele counts greater than eight and tumour variant allele counts greater than three were considered.

Genome Analysis Toolkit (v2.4, DepthOfCoverage) was used to calculate the coverage for all sequenced cases^36^. Log ratio of coverage between tumour and normal were normalized by circular binary segmentation using ExomeCNV and DNAcopy packages in R/Bioconductor. To obtain copy number alterations in tumours compared with normal tissue, GISTIC 2.0 module version 6.2 with join segment size=4 and confidence level=0.95 was used^37^. SNV and INDEL analyses were summarized into tables using R (R Core Team-2014; R Foundation for Statistical Computing, Vienna, Austria, http://www.R-project.org).

### Computational and statistical analysis

Hierarchical clustering and Kaplan–Meier analyses were performed using the stats and survival packages in R. Associations of genes with features of disease was determined using hypergeometric testing, with *P*<0.05 considered significant. Survival between two groups was determined using log-rank statistic, with *P*<0.05 considered significant. Heatmaps for mutation spectrum and pathway oncoprints were generated using the R package pheatmap. All other plots were generated using the graphics package in R.

The MutSigCV algorithm^16^ was run locally with default settings to define significantly mutated genes with input MAF files developed from the merge of MuTect and VarScan outputs. To remove any gene paralogue or alignment driven artifacts, the mutations at all positions for high-frequency genes were interrogated by BLAST against the human genome. This approach removed a number of high-frequency false positives, including CDC27 and FRG1. Mutations in significantly mutated genes and all INDELs were manually interrogated by examining BAM files in Integrative Genomics Viewer^38^. Subsequent filtering was employed were genes with at least four nonsilent mutations (>3.5%) were retained. A *P* value cut-off of 0.05 was used to obtain a list of top significantly mutated genes. Subsequent analysis of sequenced paralogues or analogous sequences was used to further restrict the results to 24 genes. A list of MutSigCV-defined genes to *P*<0.1 with associated *P* and *Q* values is provided in Supplementary Data 3. Unsupervised Random Forest clustering approach was used to cluster the pathways^39^. Random Forest package in R was used to obtain the Random forest dissimilarity measure and the data was classified into clusters using the partitioning around medoids implemented in the cluster package in R.

Clustering analysis was performed with the APC algorithm using the ‘apcluster' package in R. APC is a deterministic clustering method, which identifies the number of clusters, and cluster ‘exemplars' (that is, the cluster centroid or the data point that is the best representative of all the other data points within that cluster) from the data^40^. APC performs clustering by passing messages between the data points. It takes as input a square matrix representing pairwise similarity measures between all data points. The algorithm views each data point as a node in a network and is initialized by connecting all the nodes together where edges between nodes are proportional to Pearson correlations. The algorithm then iteratively transmits messages along the edges, pruning edges with each iteration until a set of clusters and exemplars emerges.

Two real valued messages are passed between nodes. The ‘responsibility' message computes how well-suited it is for point *i* to choose point *k* as an exemplar, given all the other candidate exemplars, *k′*, and is updated by:


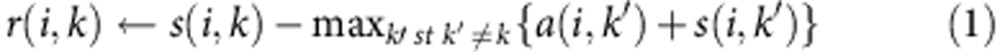


The availability message, *a*(*i*, *k*), computes how appropriate it is for point *i* to select point *k* as an exemplar, taking into account all the other points for which *k* is an exemplar, *i′*, and is given by:


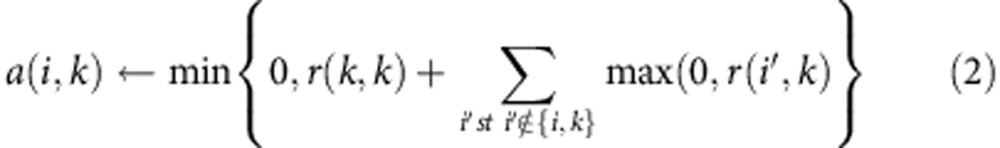


In the above equation, *a*(*i, k*) is set to the self responsibility, *r*(*k,k*), plus the sum of the positive responsibilities candidate *k* receives from other points. The entire sum is thresholded at 0, and a negative availability indicates that it is inappropriate for point *i* to chose point *k* as an exemplar and the tie is severed. The self-availability, *a*(*k,k*), reflects the accumulated evidence that point *k* is an exemplar and is updated with the following rule, which reflects the evidence that *k* is an exemplar based on the positive responsibilities sent to *k* from all points, and is updated by:


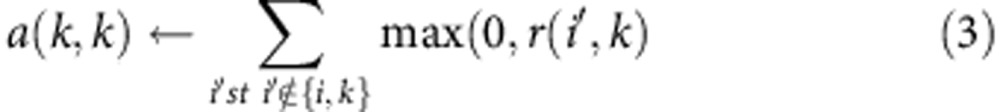


In the first iteration, all points are considered equally likely to be candidate exemplars, and *a*(*i, k*) is set to 0 and *s*(*i, k*) is set to the input similarity measure between points *i* and *k*. The above rules are then iteratively updated until a clear, stable set of clusters and exemplars emerges.

In our implementation of the algorithm, we first ran the algorithm to identify an initial set of exemplars and clusters from the data. The exemplars were then clustered together and this procedure was repeated until no more clusters emerged to identify a hierarchical structure of clusters. Networks were drawn with cytoscape^41^.

To generate mutation peg plots, protein domain information was downloaded from the Pfam database (http://pfam.xfam.org/) and isoforms were matched between data sets with Uniprot IDs. Mutation information for various TCGA tumour data sets were gathered using the cBioPortal R package, ‘cgdsr' (cbioportal.org).

To compare the mutational spectra of the PDA_COHORT data set to the TCGA tumour data sets, individual MAF files from the most current TCGA data were downloaded from the TCGA website (http://cancergenome.nih.gov/) and nucleotide substitution information was compared.

### Validation

A total of 248 nonsilent mutations from 84 of the 109 tumour/normal tissue pairs were selected to validate by Sanger sequencing. This validation encompassed 132 different genes, including most that were referenced in the text (for example *BRAF*, *BRCA2*, *RB1*, *ARID1A*). We aimed to validate mutations with allelic frequency of >0.15, recognizing that Sanger sequencing sensitivity is impaired by low mutant allele frequency. Mutations confirmation was 92% (95% CI: 87.64–94.58%). This range is consistent with other studies using MuTect to define mutations^42^, validated mutations are denoted in Supplementary Data 1.

### Immunohistochemistry and FISH

ARID1A immunohistochemistry was performed in a clinical laboratory using a BenchMark Ultra autostainer (Ventana Medical Systems). The ARID1A antibody (cat# sc-32761, Santa Cruz Biotechnology—dilution 1:150) specificity was confirmed by negative staining in cases with known mutation in ARID1A. All scores were read blind to outcome, and data were dichotomized based on loss of the homogeneous ARID1A staining observed in normal tissue or known cases with intact ARID1A gene. Association with survival was performed by Kaplan–Meier analysis. Phospho-ERK immunohistochemistry (cat#4370, Cell Signaling Technology-dilution 1:150) was performed on a DAKO autostainer. All scores were read blind to outcome or association with KRAS mutation.

Tumours from the Pdx1-Cre/CAG-βgeo-tTA/TetO-Myc transgenic mice were formalin fixed^15^, embedded and stained for haematoxylin and eosin. Immunohistochemical staining for p63 (cat#619002, Biolegend-1dilution :100) was performed on a DAKO autostainer.

Fluorecence *in situ* hybridization (FISH) at the MYC locus was performed in a CLIA clinical laboratory using Vysis LSI MYC dual colour ‘break apart' rearrangement probe. The probes detect regions directly centromeric and telomeric of the MYC genes, facilitating the detection of amplifications (both colours amplified in proximity) and/or translations (resulting in the separation of the two colours spatially).

### Functional studies

The established PDA cell lines PL45, CAPAN2, PL5, ASPC1 and HS7662 were obtained from ATCC and cultured in DMEM supplemented with 10% FBS. The cell line derived from PDA_014 was established from primary tumour fresh tissue that was digested with trypsin and plated on collagen coated plates in Keratinocyte Serum-Free Media supplemented with Bovine Pituitary Extract, EGF and 2% FBS. Tumour cells were expanded and subjected to sequencing analysis, which confirmed the retention of select tumour-associated mutations including BRAF V600E. The melanoma cell line MNT1 was cultured in DMEM supplemented with 10% FBS. Sensitivity to PLX-4032 was determined by flow-cytometry, crystal violet and CTG analysis as we have previously published^25^. For immunoblotting, 30 μg of total protein were resolved by SDS–PAGE and transferred to polyvinylidene fluoride membranes (Immobilon-P, Millipore). Membranes were immunoblotted with anti-ARID1A (SC-32761, Santa Cruz, dilution 1:500), β-actin (SC47778, Santa Cruz, dilution 1:500), anti-phospho-ERK (SC-7383, Cell Signaling dilution 1:500), anti-ERK (SC-94, Cell Signaling, dilution 1:500) and anti-horse radish peroxidase-conjugated antibodies (Jackson ImmunoResearch-dilution 1:2,500). ). Representative immunoblots are shown in Supplementary Fig. 37.

For knockdown experiments, 5,000 cells were plated in each well of 96-well plate followed by transfection of siARID1B (L-013970-01-0005, GE HealthCare/Dharmacon: pool of 3′-UGAUCAACAUGGCGGACAA-5′, 3′-CCGAAUUACAAACGCCAUA-5′, 3′-UCUCAAAGCAGACGGCAAA-5′ and 3′-ACGAGCAUCCAGAGAGAAA-5′) with transfection reagent (Lipofectamine RNAiMAX, Lif Technologies) and incubated for 4 days at 37 °C. Non-targeting siRNA pools (D001810-10-20, GE Healthcare/Dharmacon) were used as negative control. Cell Titer-Glo reagent (Promega) was used to see cell viability. About 15 μl of reagent was added to each well and mixed by orbital shaker. Luminescence of each well was measured in a microplate reader (BioTek).

## Author contributions

A.K.W., K.-U.W., M.A.W. and E.S.K. designed and directed the studies. A.K.W., K.-U.W., J.M., A.Y., M.A.C., C.J.Y., M.M. and P.M. were involved in specimens and sample collection, and logistical contribution. A.K.W., E.A.M., U.B., G.B., W.-C.L., P.K. and E.S.K. generated experimental data in the publication. E.A.M. and U.B were involved in informatics and statistical studies.

## Additional information

**Accession codes:** Tumor sequencing data is deposited at SRA (http://www.ncbi.nlm.nih.gov/sra) with the BioProject ID: PRJNA278883 and Title: Whole Exome Sequencing of Microdissected Pancreatic Cancer.

**How to cite this article:** Witkiewicz, A. K. *et al.* Whole-exome sequencing of pancreatic cancer defines genetic diversity and therapeutic targets. *Nat. Commun.* 6:6744 doi: 10.1038/ncomms7744 (2015).

## Supplementary Material

Supplementary Figures and Supplementary Tables Supplementary Figures 1-37 and Supplementary Tables 1-2

Supplementary Data 1 Data summary of SNV and INDEL.

Supplementary Data 2 Data summary of CNV

Supplementary Data 3 Summary of significantly mutated genes

## Figures and Tables

**Table 1:** Demographic and clinicopathologic features of the PDA 109 patient cohort analysed.

**Variable**	***N* (109)**
*Age (years)*	
Average (range)	66 (29-86)
	
*Gender*	
Male	55 (50%)
Female	54 (50%)
	
*Diagnosis*	
PDA	94 (86%)
Adenosquamous	11 (10%)
Colloid	4 (4%)
	
*Grade*	
1	5
2	77
3	27
	
*Survival (months)*	
Median	21

PDA, pancreatic ductal adenocarcinoma.

**Table 2:** Tabular summary of potential pharmacological strategies for specific genetic pathway deregulation.

	**Genetic event (frequency)**	**Target: drugs**
BRAF	BRAF V600E (3%)	BRAF: Vemurafinib
PIK3CA	PIK3CA activating mutation (1%)	PIK3CA: BKM120, GDC0941
RB pathway	CDKN2A loss (41%) CDK4 amp, CCND1 amp. (9%)	CDK4/6: PD-0332991, LEE-11CDK4/6: PD-0332991, LEE-11
Myc	Myc amp (14%)	CDK9: PHA767491BET-Bromodomain: JQ1
Double-strand break repair	FANC gene family (14%)BRCA gene family (3%)	DNA: Mitomycin CPARP: Olaparib
Beta-Catenin	RNF43 loss (7%)AXIN1/2 or APC loss (11%)	Porcupine: LGK974Tankyrase: XAV939
Notch	Notch family amp (31%)	Gamma secretase: MK0752
