# Supplementary material for: Whole-exome sequencing of pancreatic cancer defines genetic diversity and therapeutic targets
Source: Nat Commun. 2015 Apr 9;6:6744. doi: 10.1038/ncomms7744 (PMC4403382; doi:10.1038/ncomms7744)
Supplement: Supplementary Figures and Supplementary Tables — Supplementary Figures 1-37 and Supplementary Tables 1-2 [file ncomms7744-s1.pdf]

Supplementary Figure 1

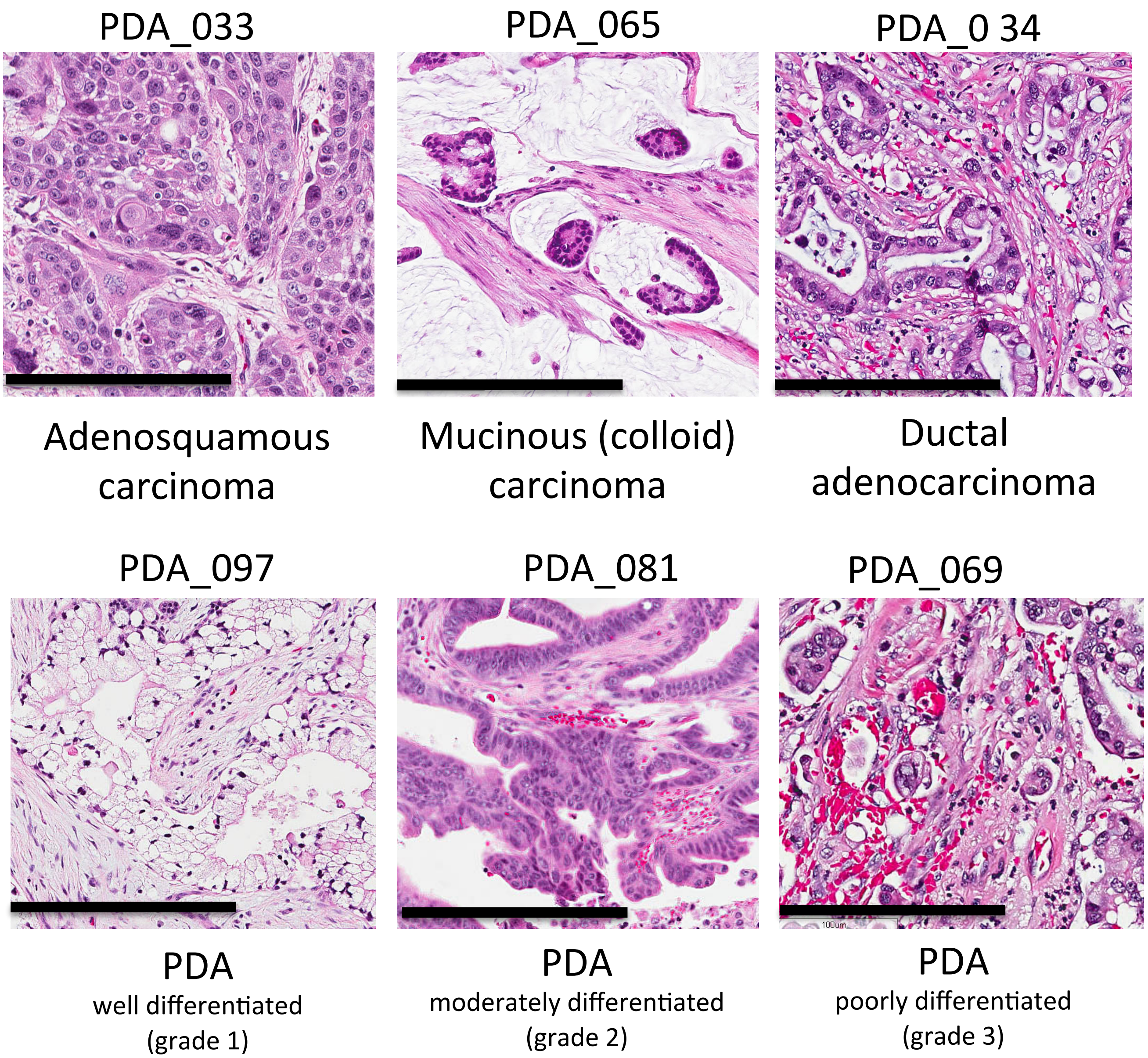

Different histological forms and grades of PDA in the sequenced cohort Representative hematoxylin/eosin staining of adenosquamous, mucinous, and ductal pancreatic adenocarcinoma no special type employed in the study. Different pathological grades of PDA in the sequenced cohort (Scale bar is 200 μm).

Supplementary Figure 2

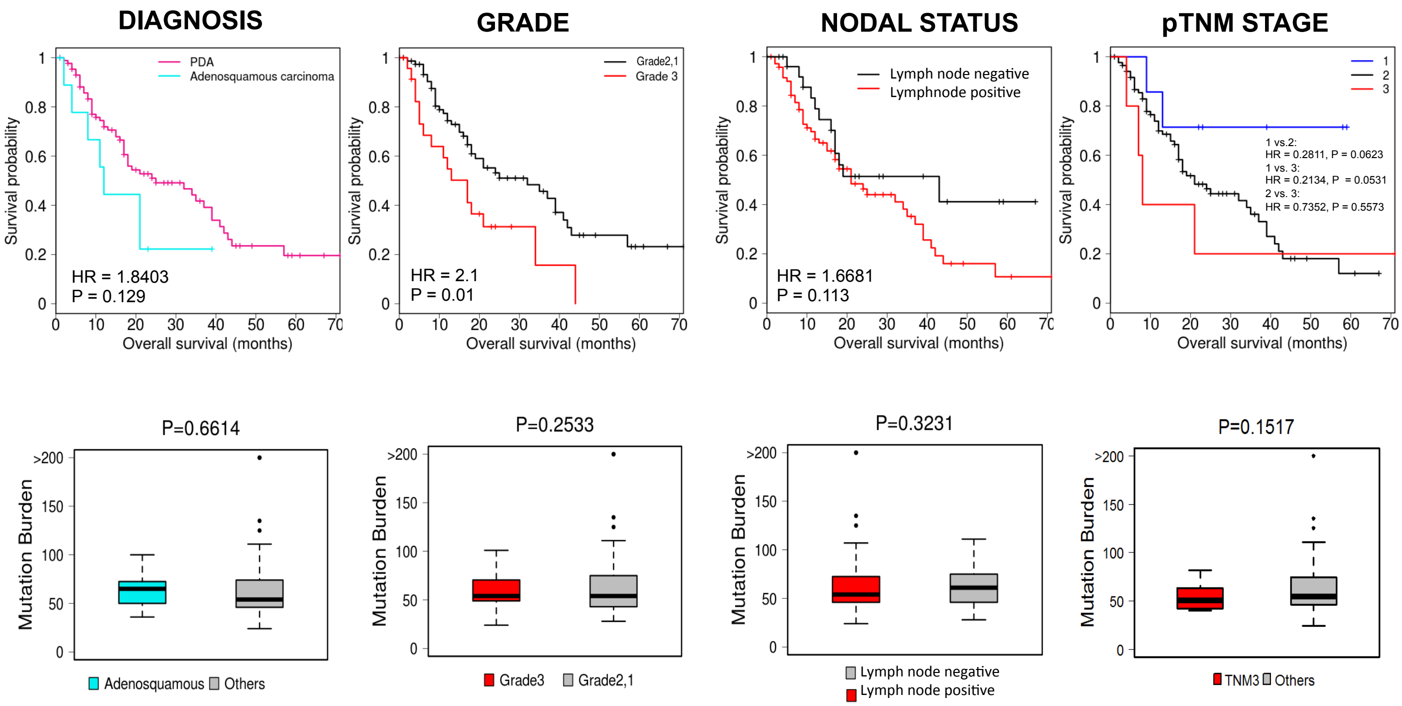

Association of clinical/pathological features with disease outcome and mutation burden in the sequenced cohort: The histological form of PDA, tumor grade, nodal status, and pTNM stage were evaluated for their individual association with overall survival, HR and P-value were obtained from Cox proportional hazard test. Tumor grade 3 was significantly associated with poor outcome, while adenosquamous histology, pTNM stage, and node-positive disease trended toward poor outcome. Mutation burden was not associated with any of these features of PDA. The boxes show the distance between the first and third quartile with the whiskers extending up to 1.5 times the interquartile range, p-value was determined by Student's t-test.

Supplementary Figure 3

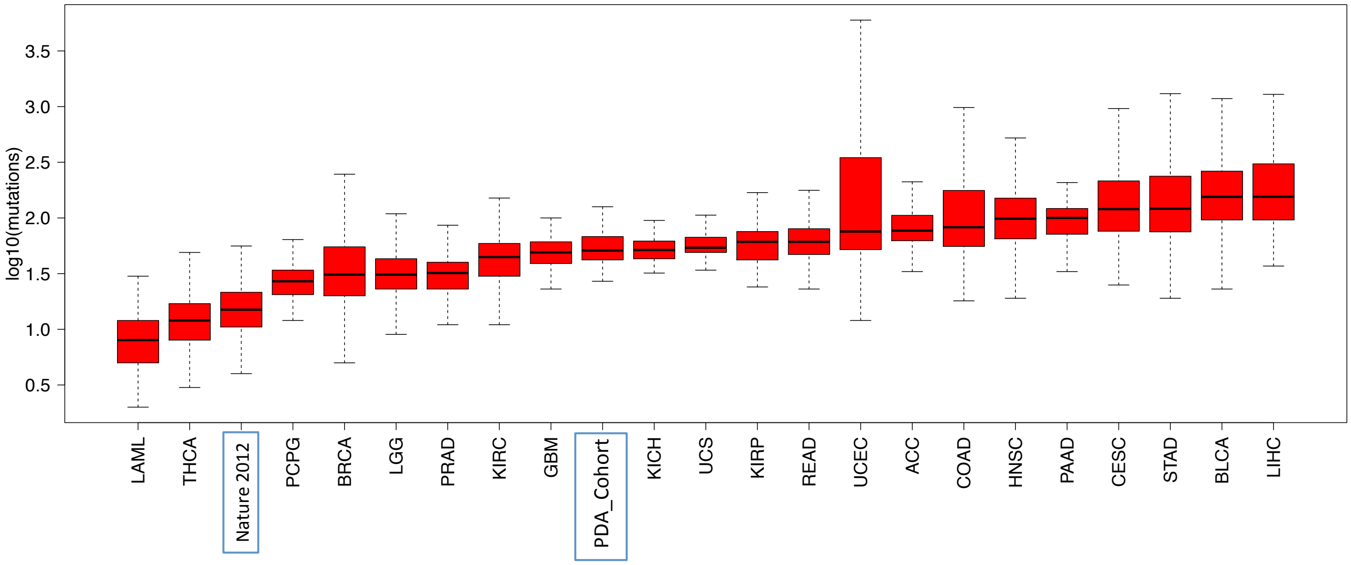

Comparison in mutation frequency across different studies: The average mutation frequency per case is plotted across multiple different studies. All data are from published TCGA cohorts. The Nature 2012 study has 99 cases, and the present PDA_Cohort has 109 cases. The Nature 2012 study exhibited a relatively low average mutation burden per case (26 mutations). In the present study an average of 67 mutations per case were identified, which is consistent with other solid malignancies including colorectal cancer, kidney cancer and prostate cancer.

Supplementary Figure 4

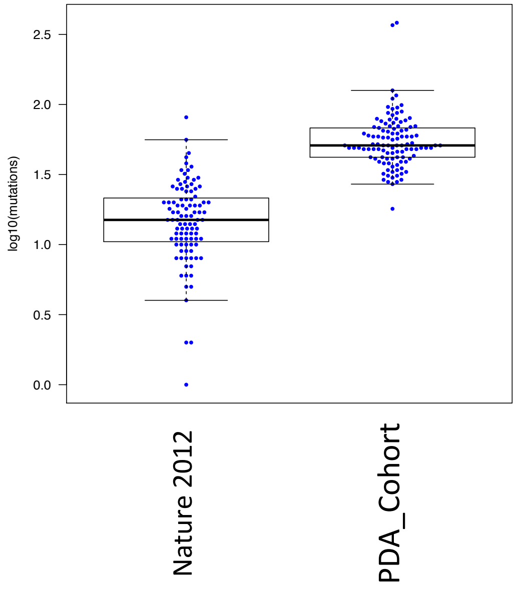

Comparison in mutation frequency across different PDA studies: Direct comparison of mutational burden with the Nature 2012 study of 99 patients. The boxes show the distance between the first and third quartile with the whiskers extending up to 1.5 times the interquartile range.

Supplementary Figure 5

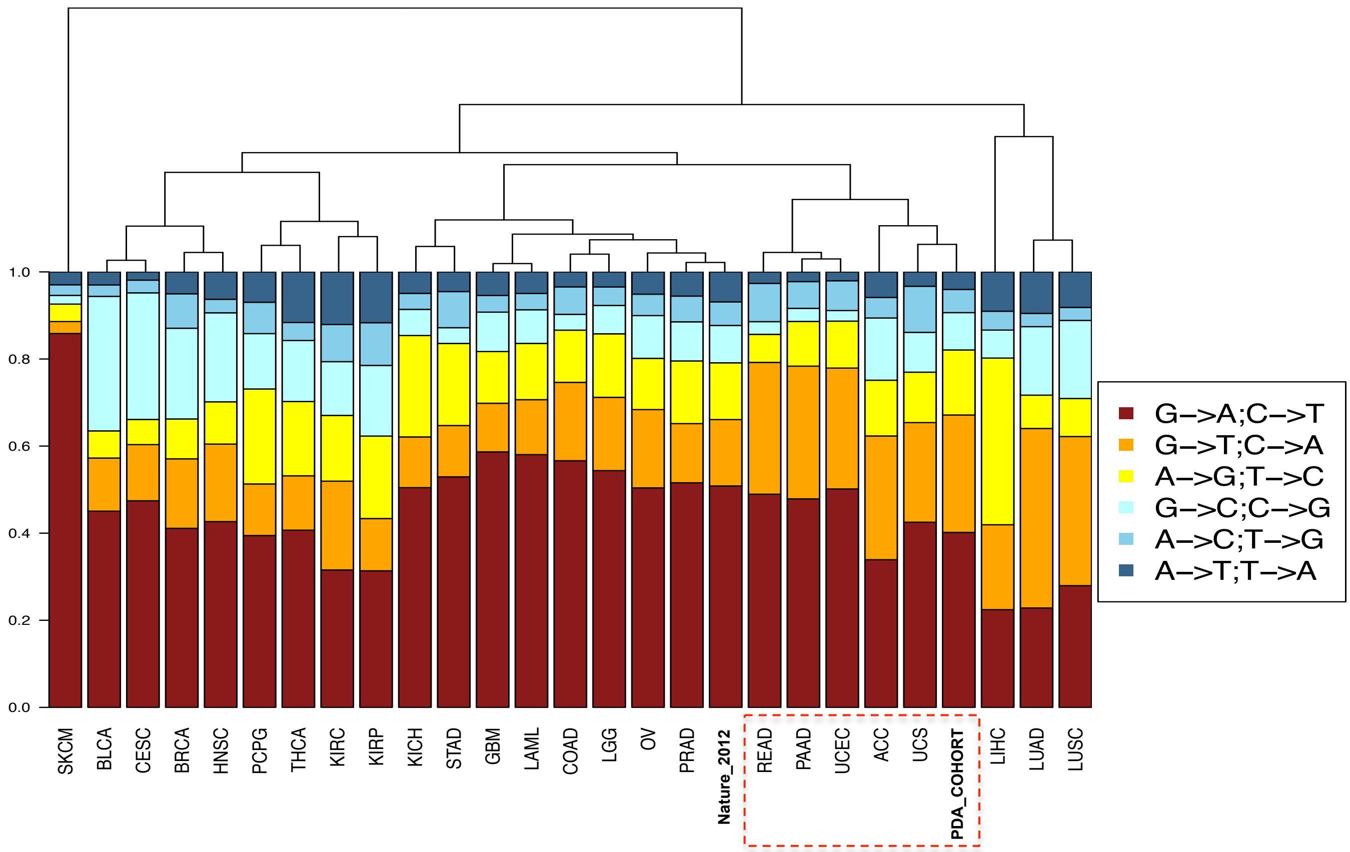

Clustering of PDA cohort vs. cohorts sequenced by the TCGA: The mutation spectra observed in the sequenced cohort (PDA_COHORT) was evaluated relative to sequencing of multiple other cancers by unsupervised clustering based on Euclidean distance. The sequenced cohort clustered in a branch that includes TCGA adenocystic carcinoma (ACC), uterine carcinocsarcoma (UCS), endometrial carcinoma (UCEC), rectal adenocarcinoma (READ), and the pancreatic adenocarcinoma (PAAD).

Supplementary Figure 6

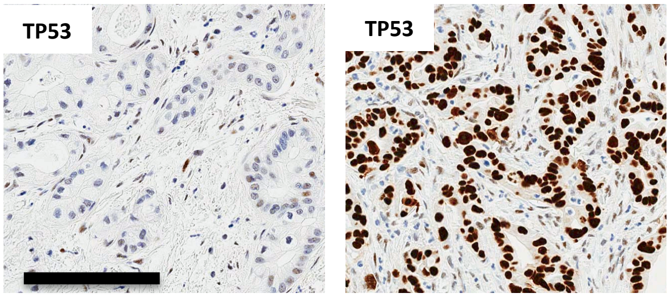

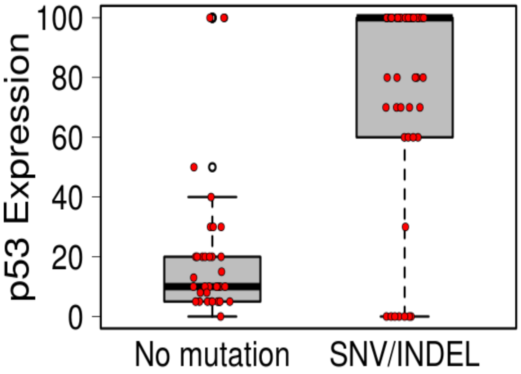

R=0.5244
P=4.256E-07

TP53 mutation strongly associates with the accumulation of protein: Of the 109 sequenced cases 84 were stained for the protein levels of TP53 by immunohistochemistry. Representative staining is shown (scale bar 100 µm). The percentage of tumor nuclei staining positive was determined by a pathologist blinded to the mutation status of the cases. The data were subsequently stratified based on the mutational status of TP53, that exhibited a strong positive correlation between positive staining and mutation. The boxes show the distance between the first and third quartile with the whiskers extending up to 1.5 times the interquartile range. Correlation co-efficient and p-value were obtained from Spearman correlation test.

Supplementary Figure 7

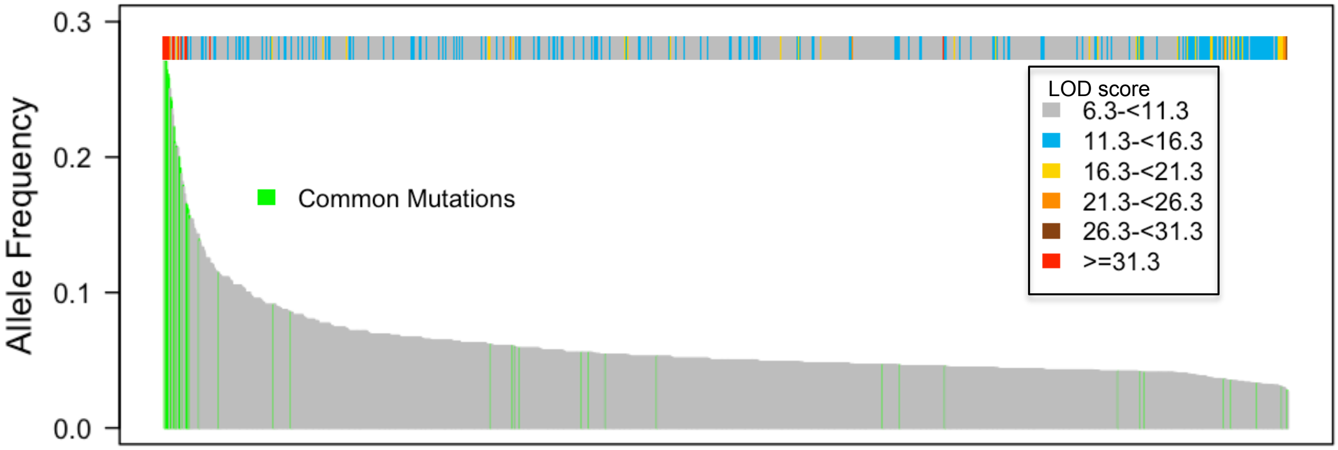

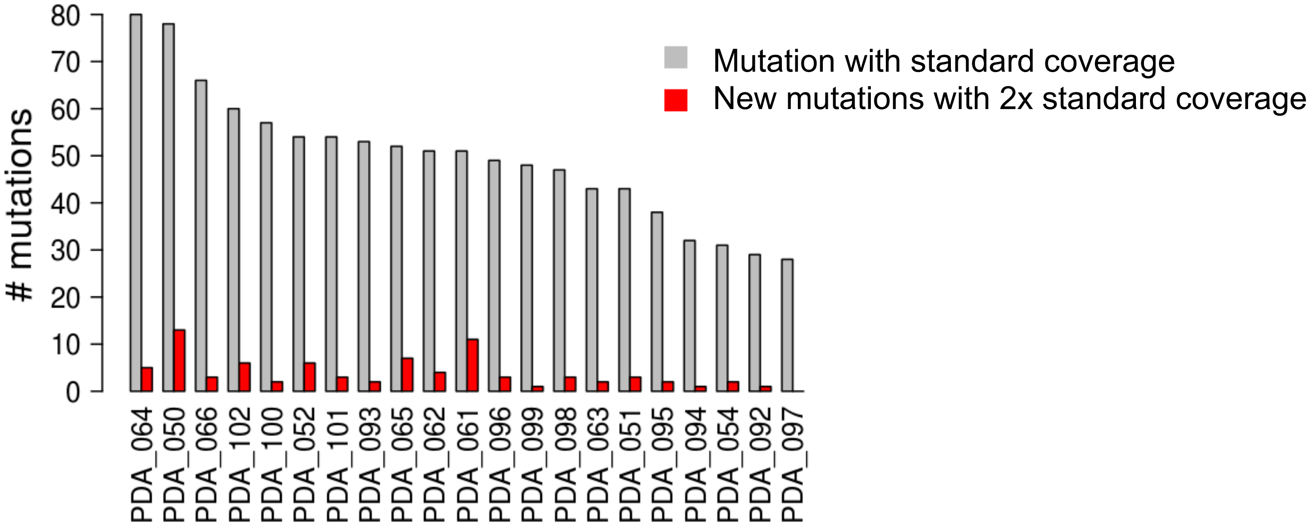

Comparison with deeper sequencing: Mutations were called using Mutect across 21 cases sequenced to ~52x or ~120x coverage depth. Allele frequency and LOD score is shown for mutations identified at either both depths (green) or only with deeper sequencing (gray). When statistical cutoffs are applied, relatively few genes are identified through the deeper sequencing across the 21 cases.

Supplementary Figure 8

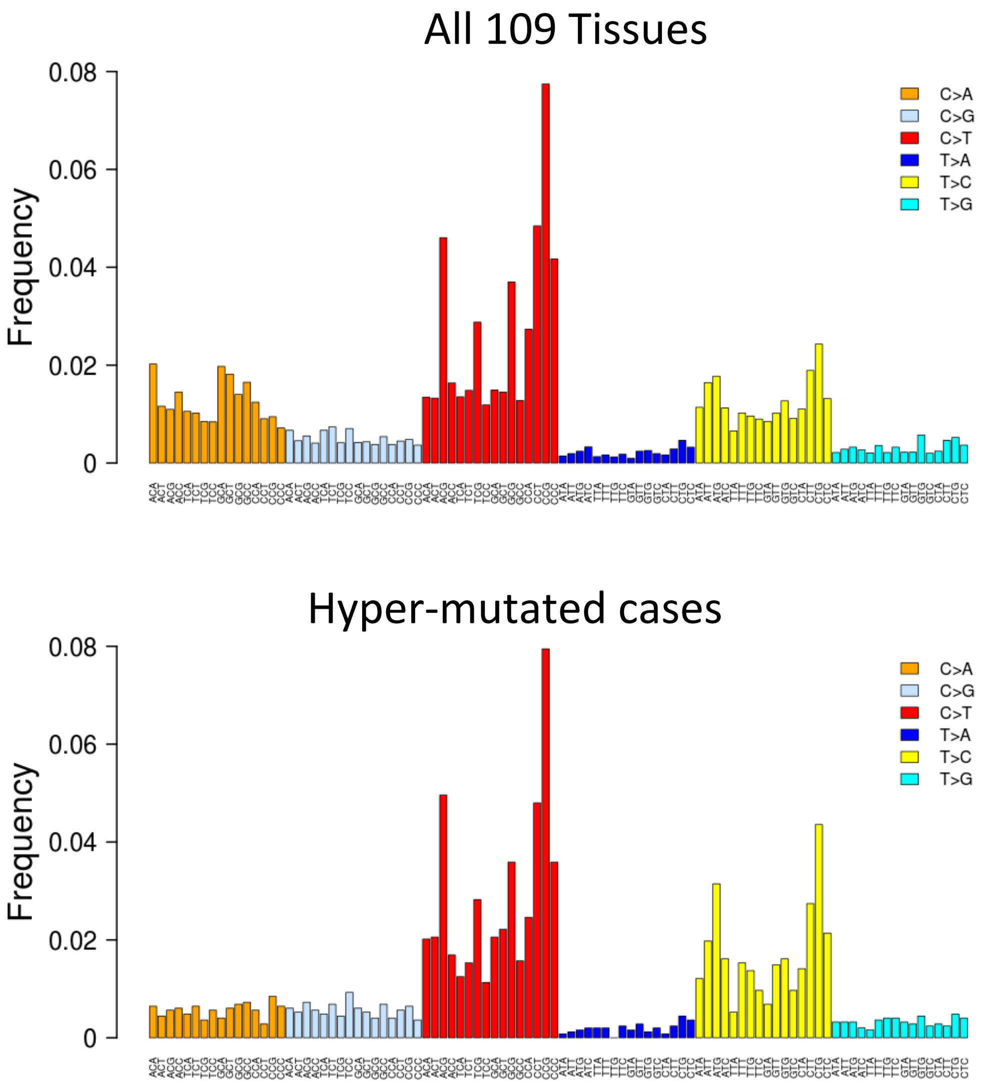

Tri-nucleotide target spectrum of mutation in PDA and hype-rmutated cases: The overall spectrum of mutations within the entire cohort (109 tissues). The dominant pattern of C>T transversions falls into mutation spectrum signature class 1B (Alexandrov et al. 2013), mutations at CpG tri-nucleotides associated with aging. However, there is significant C>A mutation consistent with smoking contributing to overall mutation burden. The T>C mutation spectrum observed in top mutated cases (lower histogram) reflects a high requency of mutation at CTG tri-nucleotide that is consistent with deficits in mismatch repair.

Supplementary Figure 9

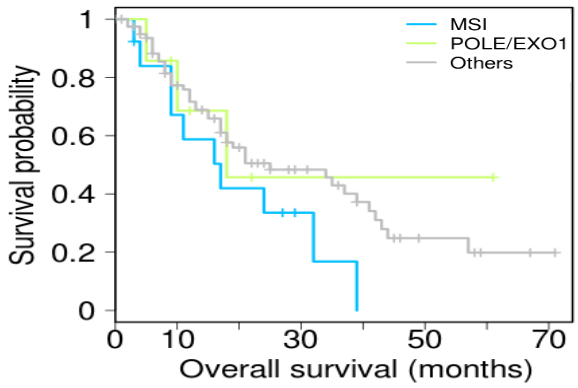

GROUP	N	HR	P-value
MSI	13	1.8109	0.0947
POLE/EXO1	7	0.8245	0.7468
Others	82		

Survival based on the presence of genetic lesions in genes associated with mutator phenotypes. Assessment of mutations or homozygous deletion on mismatch repair (MSH2, MLH1, PMS2, MSH6, MSH3) or other processes driving high mutational burden (POLE, EXO1) on overall survival. HR and P-values were obtained from Cox proportional hazard test.

Supplementary Figure 10

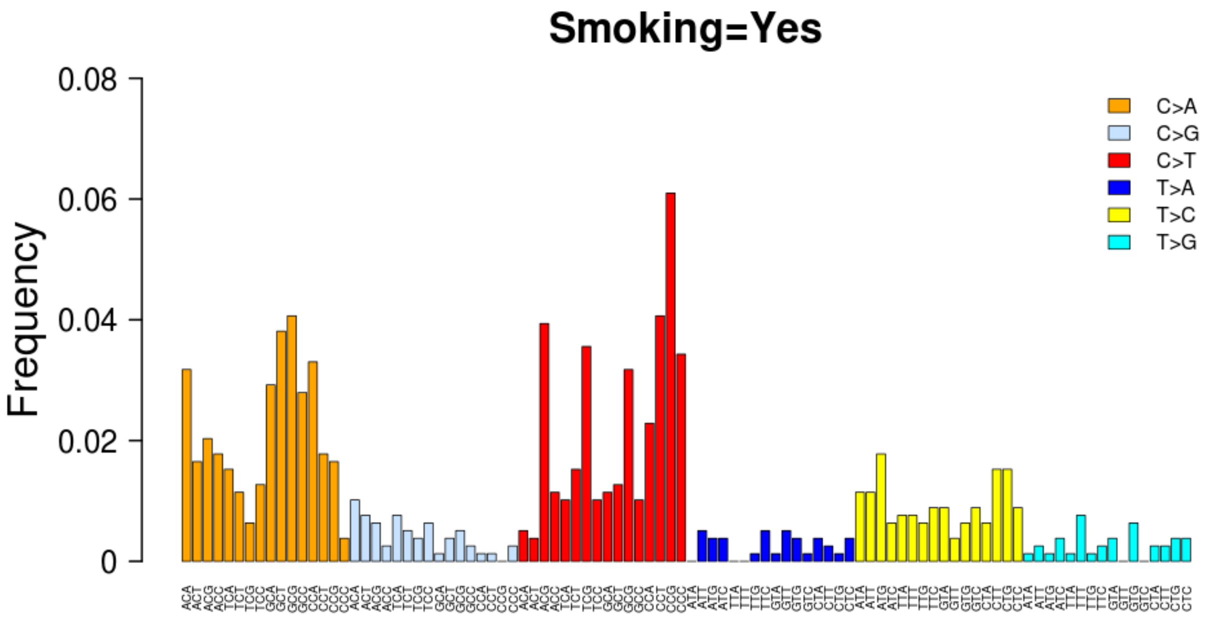

Mutation spectrum and smoking: The overall spectrum of mutations for PDA arising in smokers is shown. The increased level of C>A transversions is consistent with mutation spectra for smoking in bladder cancer and squamous cell cancer of the head and neck, which have a similar increased risk of malignancy with smoking.

Supplementary Figure 11

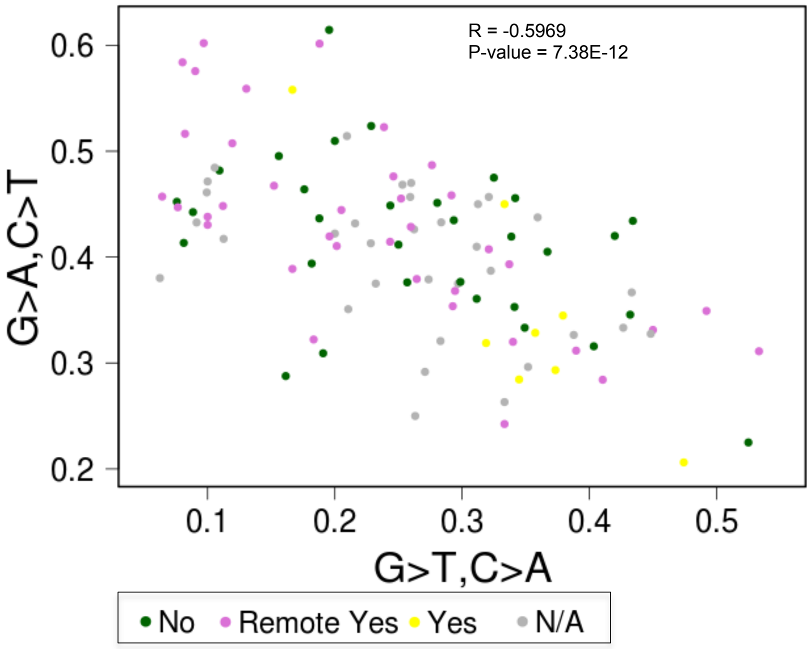

Mutation transversion/transitions and smoking: PDA associated with smoking status exhibited a particular elevation in the ratio C>A to C>T. Correlation co-efficient and p-value were obtained by Pearson correlation test.

Supplementary Figure 12

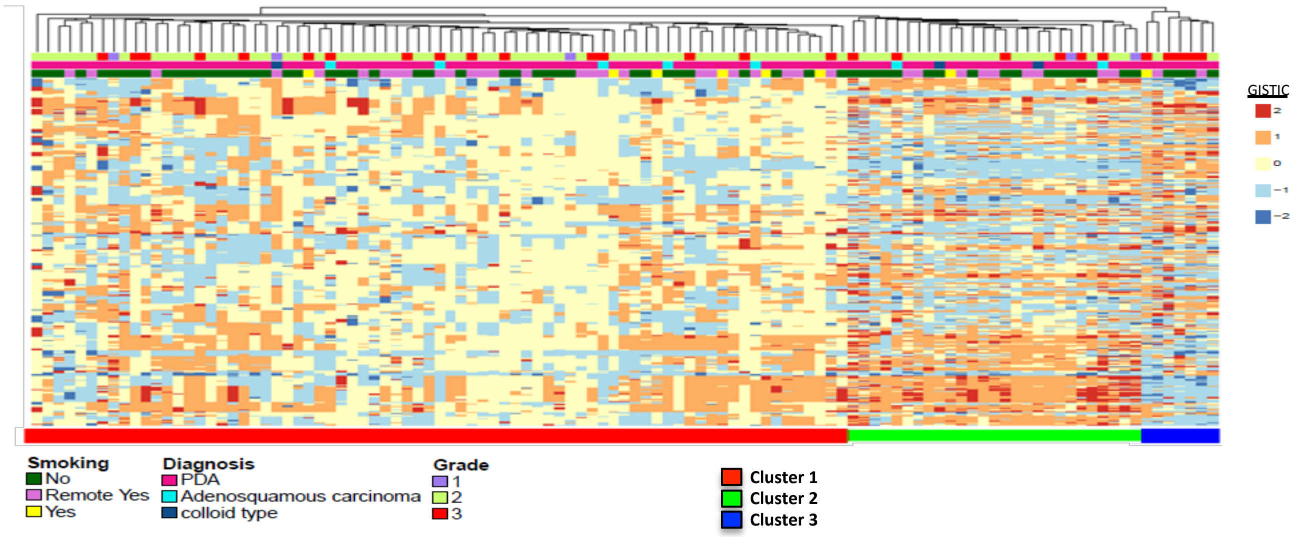

Euclidean distance based clustering of copy number variation: The gene level CNV as determined using GISTIC2.0 was clustered in chromosome order based on Euclidean distance. Three predominant branches emerged with higher levels of chromosomal aberrations in clusters 2 and 3. Select CNV alterations differentiated between cluster 2 and 3.

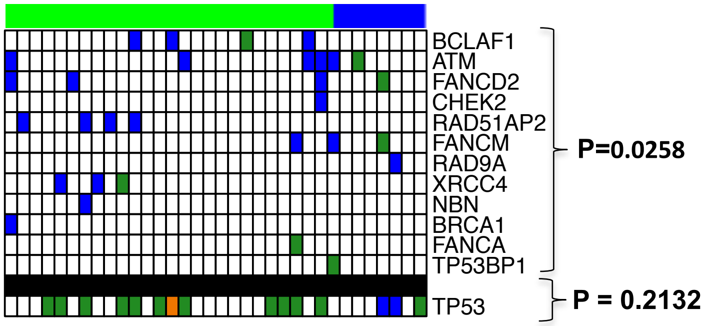

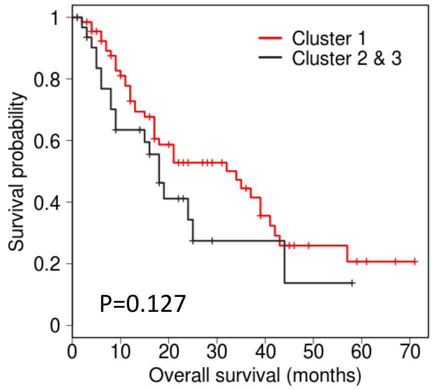

Association of mutations in DNA damage repair pathway with copy number variation: Mutations in known genes involved involved in DNA damage response and repair were evaluated for their association with CNV cluster 2 and 3 using a hypergeometric test. There was an overall enrichment for these genes in the clusters with more chromosomal alterations. In contrast, mutation/loss of TP53 was not associated with the level of copy number variation. Survival analysis by CNV: The overall survival of cluster 1 vs. clusters 2 and 3 was determined by Kaplan-Meier analysis. Clusters 2 and 3 trend toward poor outcome. HR and P-value were obtained from Cox proportional hazard test.

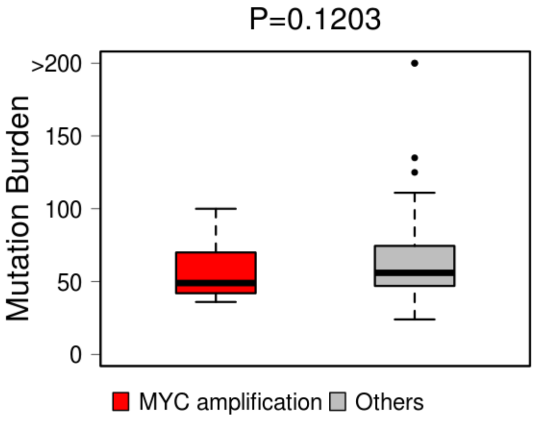

MYC Amplification and genetic events		
**Gene**	**P-Value**	**OR**
TP53	0.5562	1.6603
SMAD4	0.125	3.0837
KRAS	0.5956	-
CDKN2A	0.5422	1.51

MYC Amplification and pathological features		
**Feature**	**P-Value**	**OR**
Nodal Status (0 vs. 1)	0.7427	0.7939
Grade (3 vs. others)	0.0834	3.0235
Adenosquamous (yes vs. no)	0.0005	12.8915
TNM Stage (3 vs. others)	0.1571	3.9652

Features of MYC amplified pancreatic cancer: The mutation burden of tumors with MYC amplification was determined, and shows no significant impact of MYC amplification on mutation burden (p-value was determined by Student's t-test). Similarly, no significant association was determined relative to established genetic events in PDA. In the analysis of MYC amplification with pathological features of PDA there was strong association with the adenosquamous subtype of PDA (p-values and odds ratios were determined by Fisher's exact test).

Supplementary Figure 15

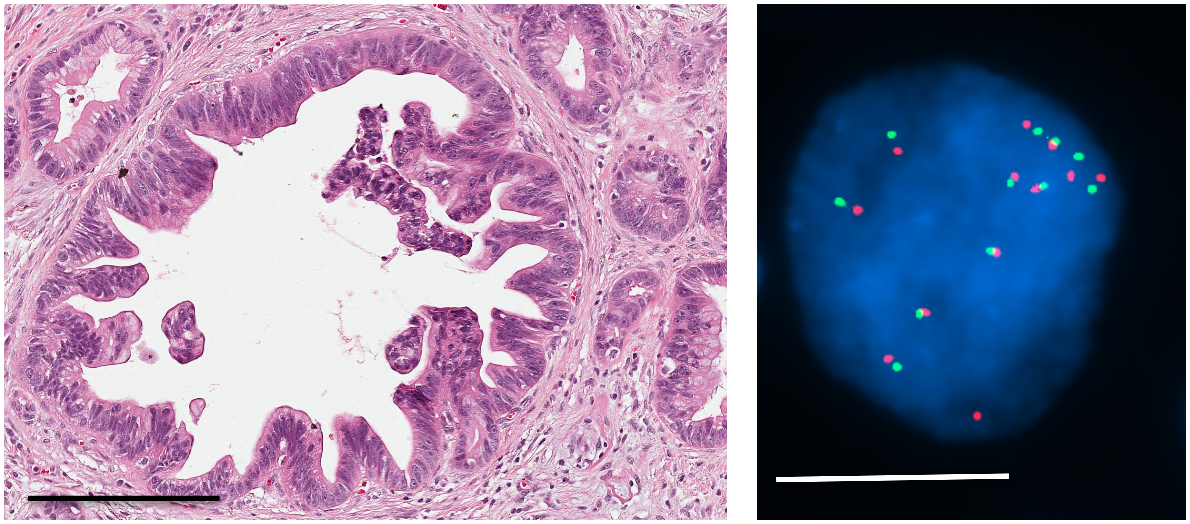

Increased MYC copy number in PanIN lesions of adenosquamous PDA: A PanIN 3 lesion in a case that gave rise to adenosquamous PDA was analyzed for MYC copy number alterations by FISH. The PanIN lesion exhibits gene amplification. Scale bar for left panel is 400 µm and right panel is 5µm.

Supplementary Figure 16

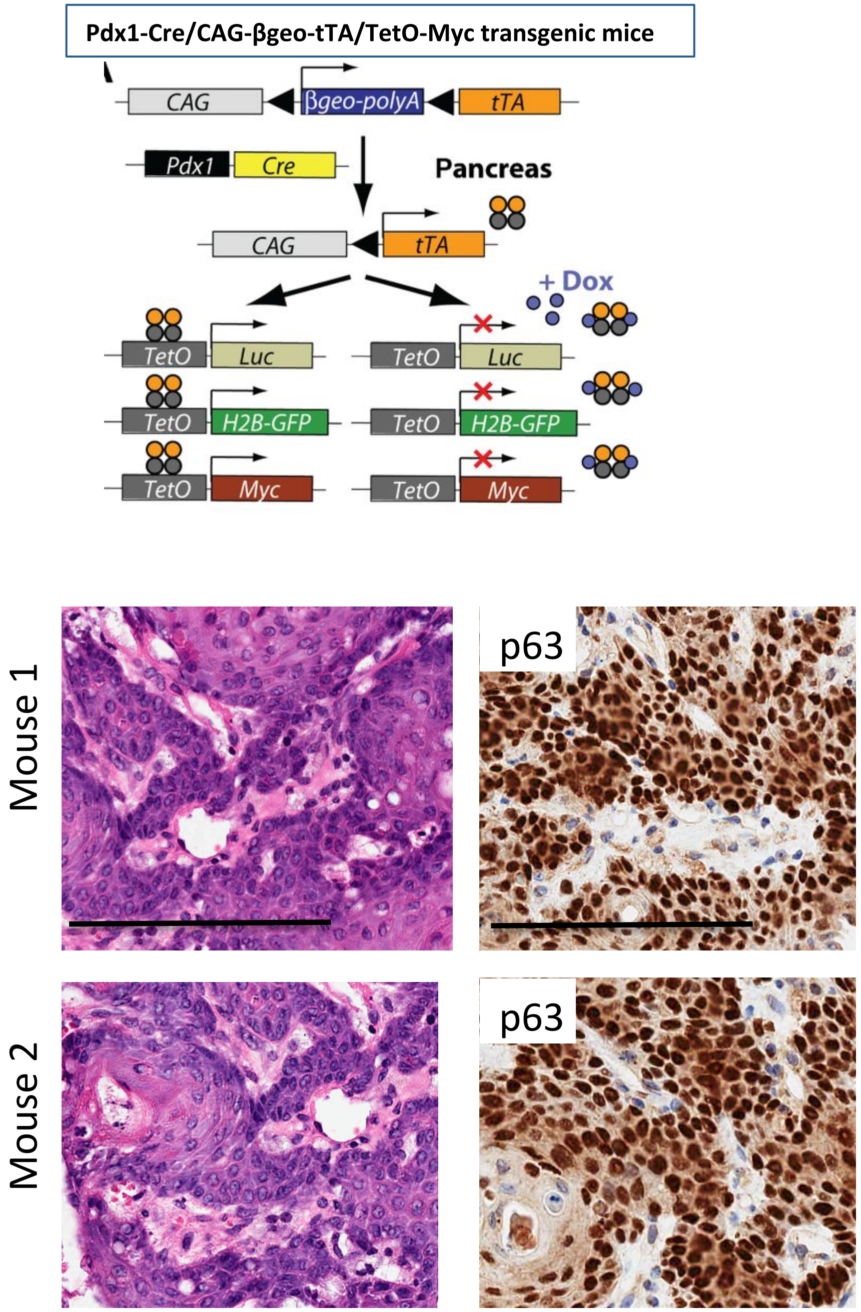

MYC driven pancreatic cancers have a adenosequamous histology and marker expression: The Pdx1-Cre/CAG-βgeo-tTA/TetO-Myc transgenic mice (schematic as from Lin et al., 2013 *Cancer Research)* develop pancreatic neoplasms with a short latency. The tumors that arise in this model exhibit a histology consistent with adenosquamous pancreatic cancers. Furthermore the tumors express p63 which is an established marker of squamous differentiation (Scale bar is 100 µm).

Supplementary Figure 17

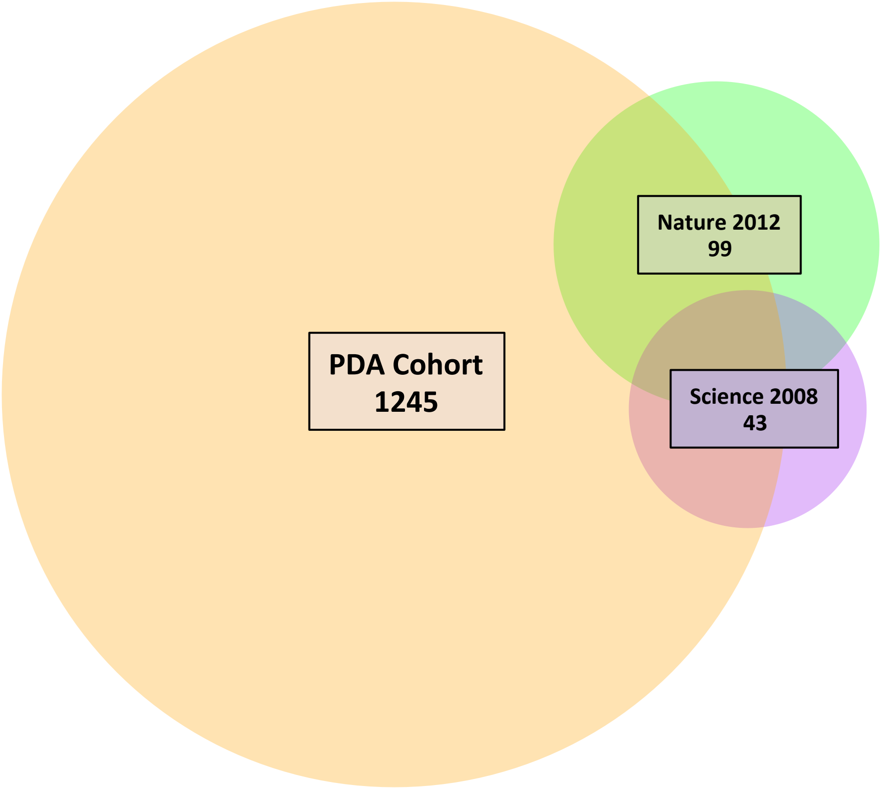

Euler plot of the number of recurrent genes and overlap with published analysis of PDA: A Euler plot comparison the number of recurrent mutations vs. two published studies. Nature 2012 (Biankin et al.) represents 99 clinical cases of PDA, Science 2008 (Jones et al.), is composed of 24 xenografts or cell lines derived from primary tumors. The total number of recurrent mutations (n>1) is indicated as is the overlap between studies.

Supplementary Figure 18

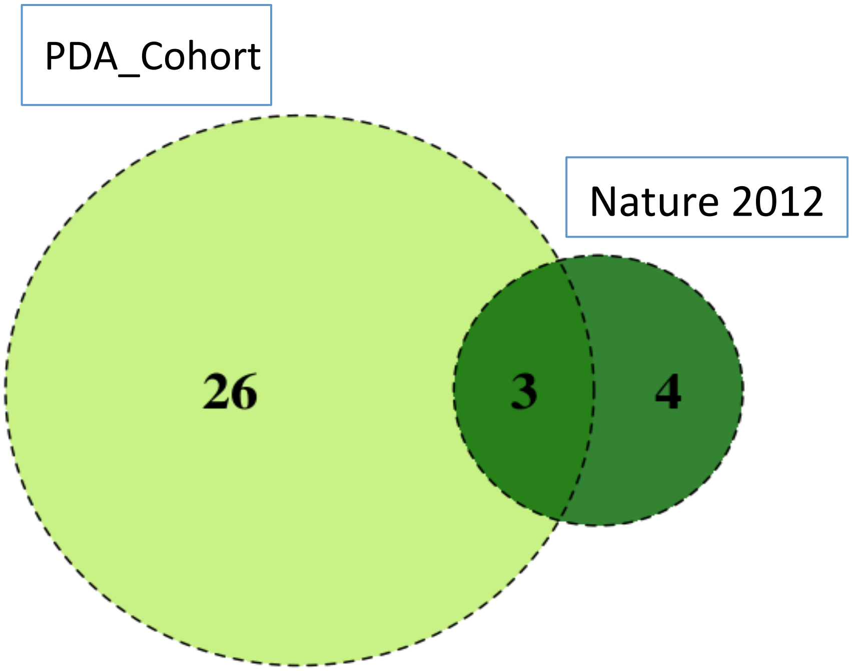

Nature, 2012

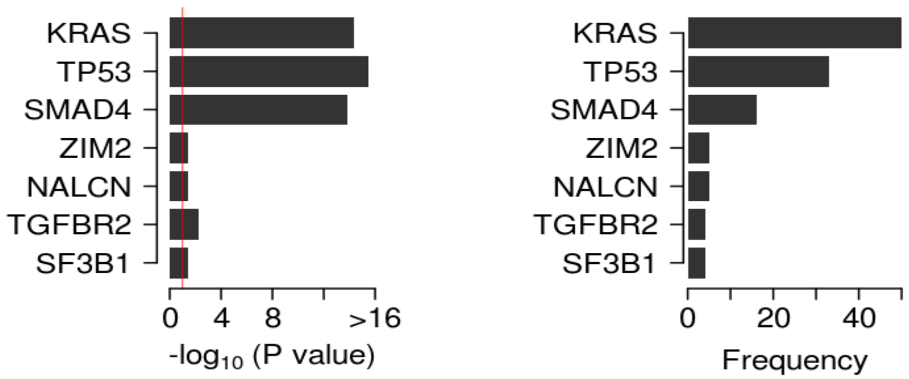

Venn diagram of significantly mutated genes: A Venn diagram of significantly mutated genes defined with the MutsigCV algorithm ($p<0.05$ and recurrence frequency >3.5%). The cohort from Nature 2012 contains 99 cases, and the current study cohort contain 109 PDA cases. The three genes in common are KRAS, TP53 and SMAD4.

Supplementary Figure 19

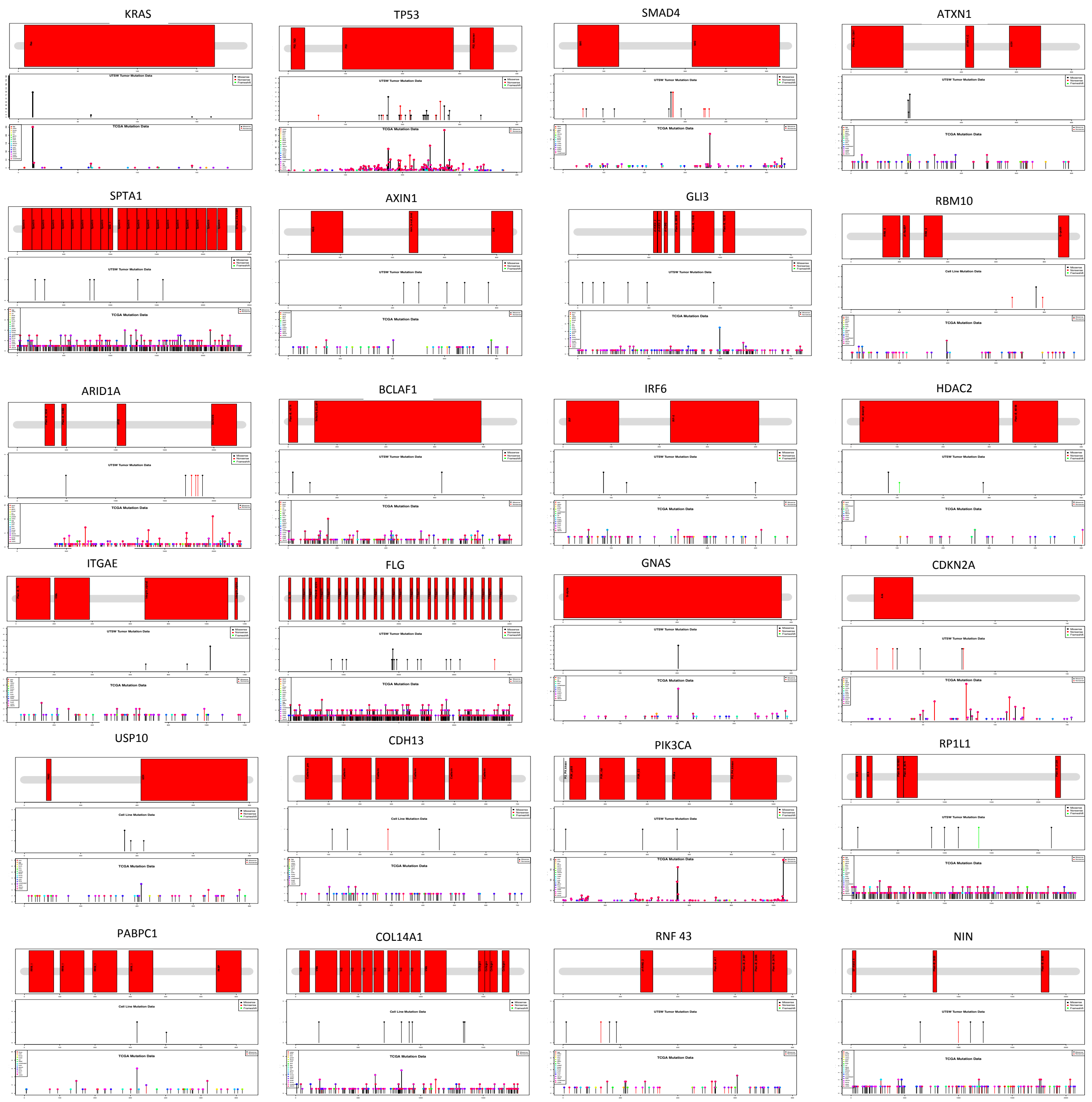

PEG plots of significantly mutated genes: PEG plots show the domain structure and location of the mutations defined in the current study. Lower PEG diagram depicts the location of mutations as defined across all TCGA sequenced cases.

Supplementary Figure 20

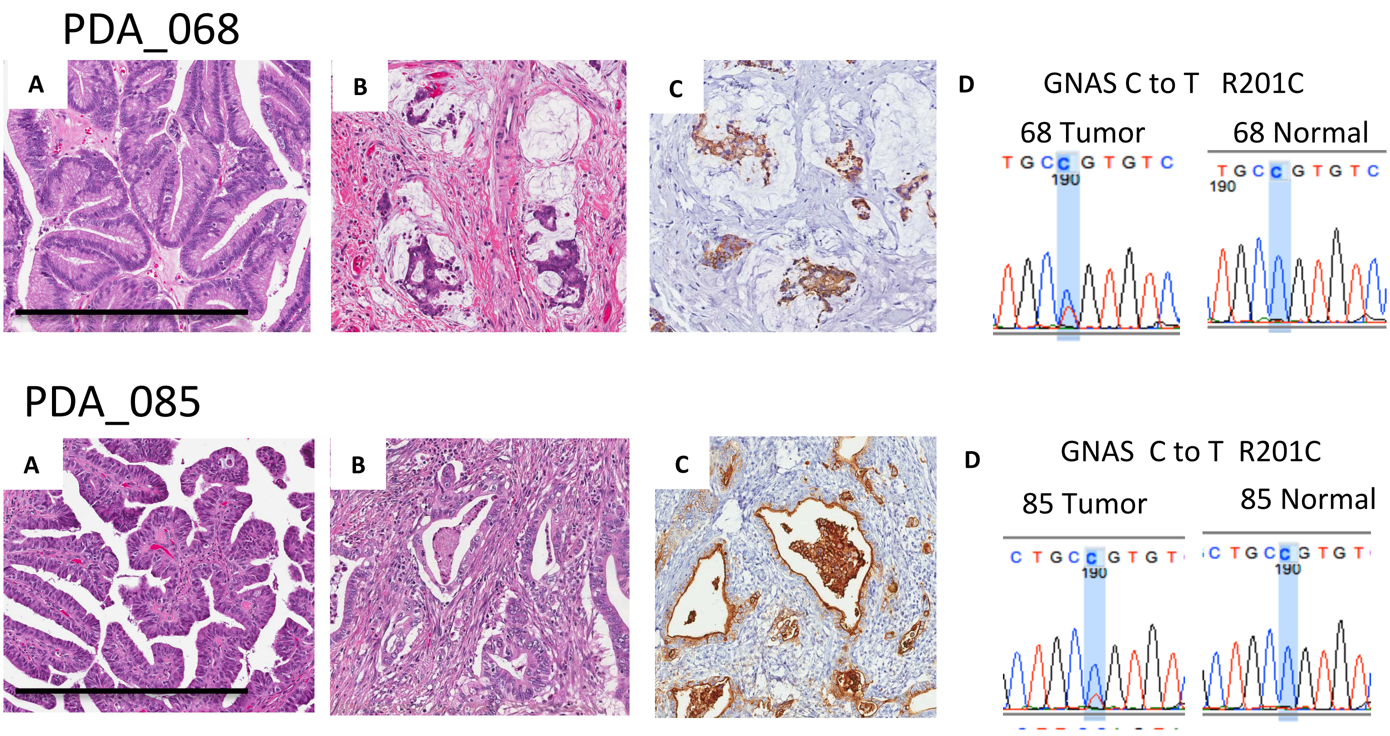

GNAS mutations in histological types of PDA: GNAS R201C mutation in mucinous carcinoma (PDA_068) arising from IPMN. (A) IPMN, intestinal type. (B) Mucinous carcinoma. (C) MUC2, intestinal type mucin stain, in mucinous carcinoma. (D) Sanger sequencing of GNAS mutation GNAS R201C mutation in ductal carcinoma NOS (PDA_085) arising from IPMN. (A) IPMN, pancreaticobiliary type. (B) Ductal carcinoma, NOS. (C) MUC1 positivity. (D) Sanger sequencing of GNAS mutation. Scale bar is 200 µm for all images, except PDA_085 panel (A) which is 100 µm.

PDA_071

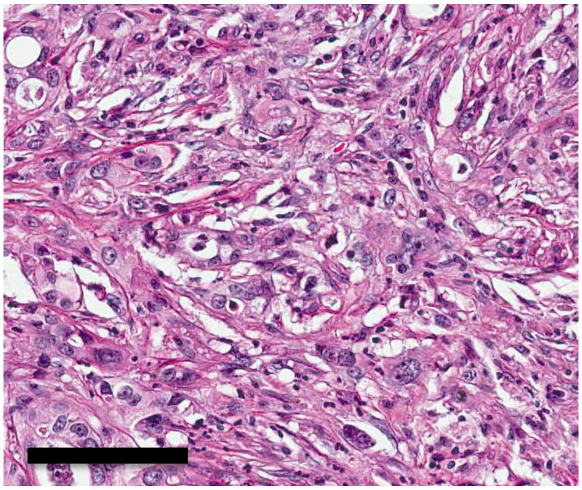

PDA_0 81

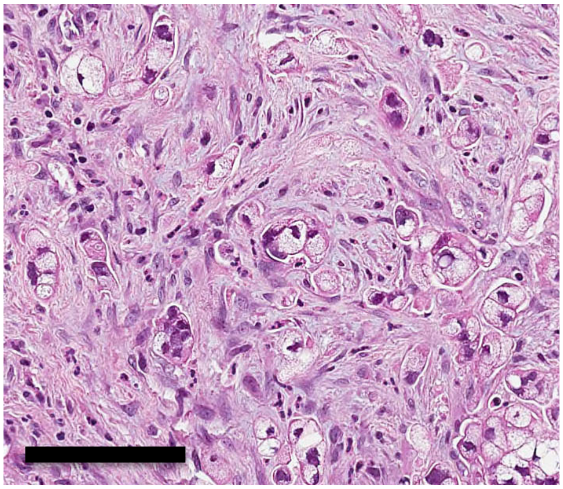

Histology of PDA cases with RBM10 mutations: Case PDA_071, pT3 N1, poorly differentiated carcinoma alive with no recurrent disease at 30 months follow-up. Case PDA_081, pT3N1, moderately to poorly differentiated carcinoma alive with no recurrent disease at 46 months follow up. Scale bar is 100 µm.

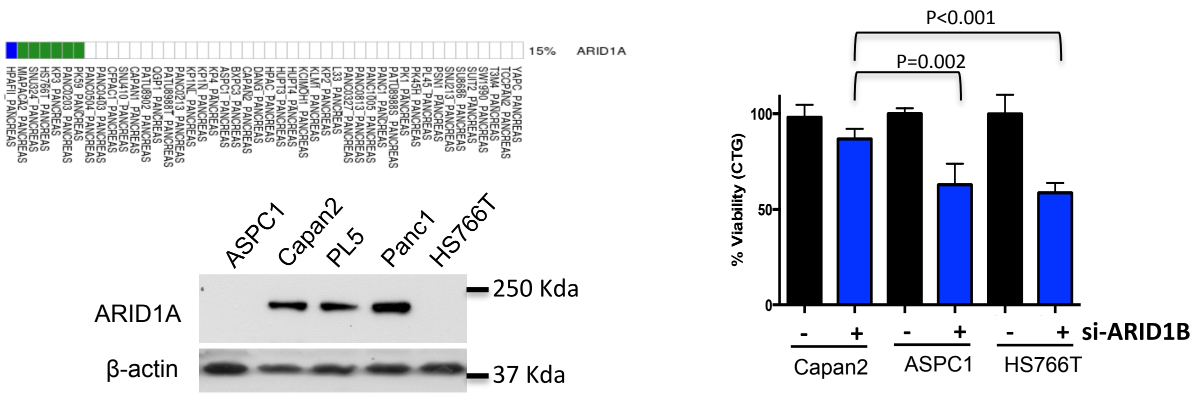

Impact of ARID1B depletion on ARID1A-deficient pancreatic cancer cell lines: 15% of PDA cell lines harbor genetic deficiency of ARID1A, and protein levels are observed to be diminished in multiple models. Cells deficient in ARID1A are selectively sensitive to ARID1B knockdown. Error bars show standard deviation and p-values are by Student's t-test.

Supplementary Figure 23

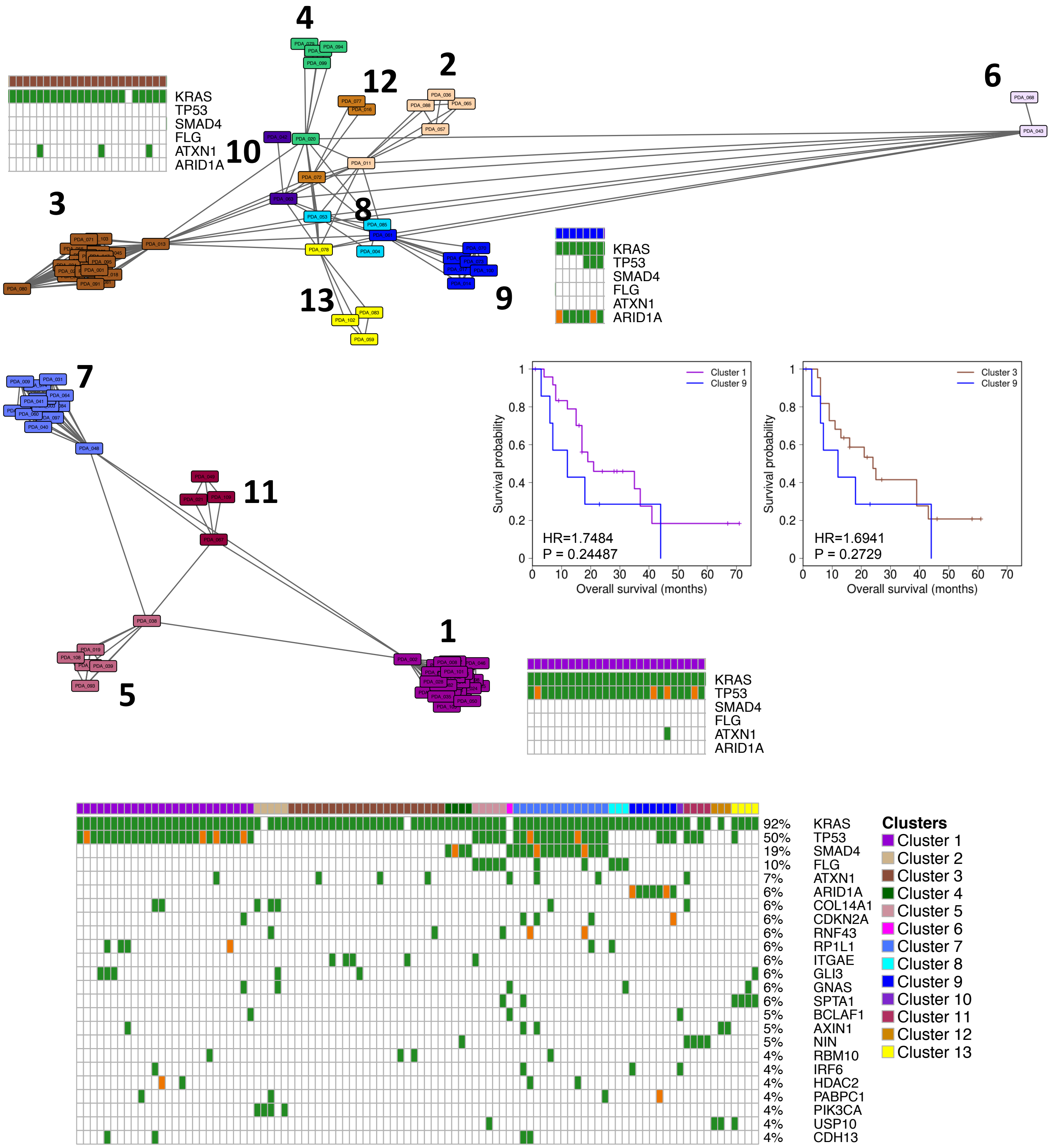

APC clustering of significantly mutated genes: The collection of MutsigCV significant genes were subjected to APC clustering to generate a network. Oncoprints for select networks are shown, as is a heatmap describing all clusters. The association of a pair of networks with survival are shown.

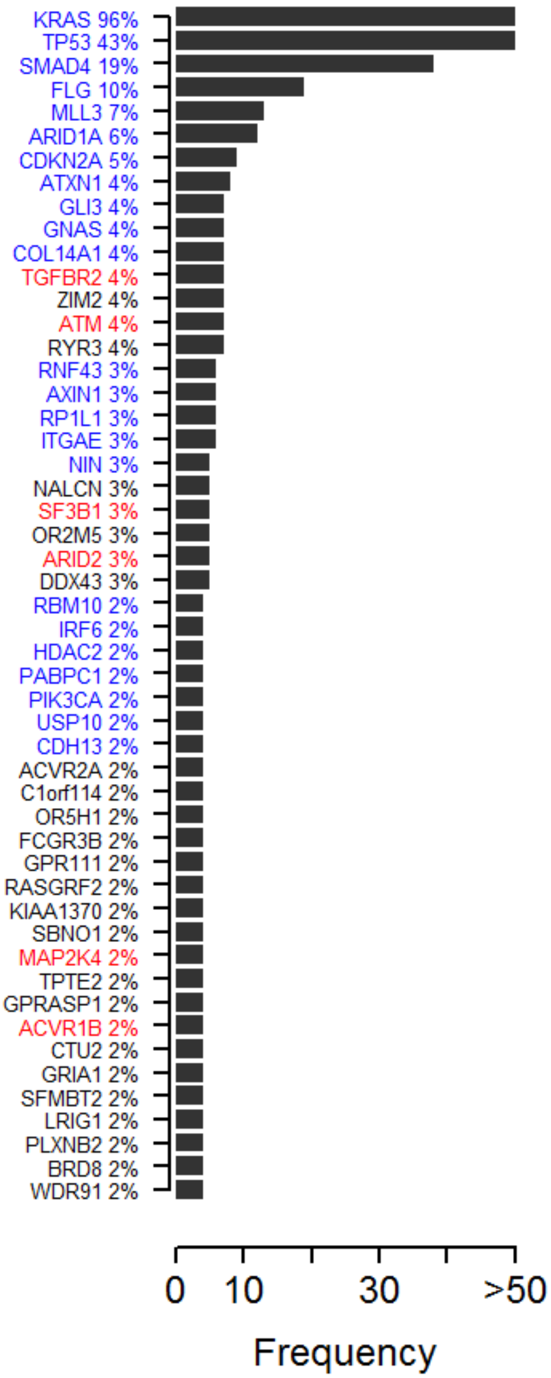

Meta-analysis of sequenced cases defines additional significantly mutated cases: The MutsigCV algorithm was applied to combined data from the current study and Nature 2012 totaling 208 cases to identify significantly mutated PDA genes. Cutoffs were relaxed to accept recurrence frequency of >1.8%, all genes pass a statistical cutoff of $p<0.05$. Genes denoted in blue are from the current study, genes in red are "cancer genes" that are defined in the combined dataset, genes in black are additional "significant" genes in the combined dataset.

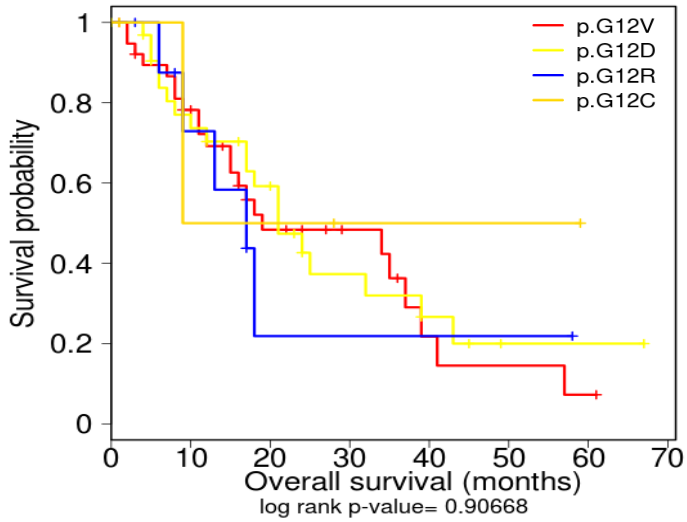

Association of G12 mutant alleles with survival: The association of the indicated codon 12 mutations with overall survival was determined by Kaplan-Meier analysis. There was no significant association between the individual G12 alleles in reference to survival (p-value was determined by Cox proportional hazards test).

PDA_051

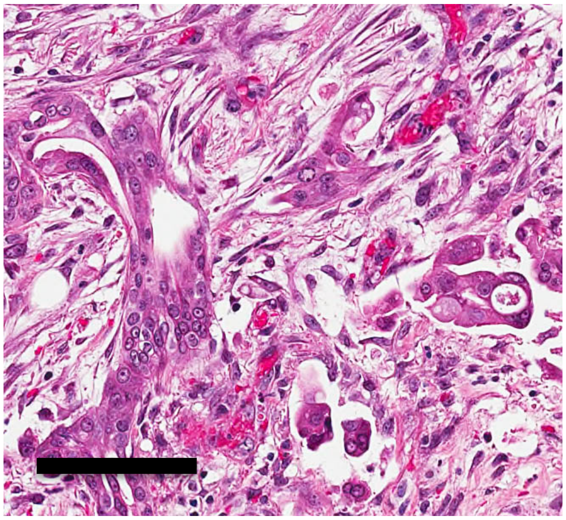

Case	Stage	Grade	Survival months	Vital Status	Resection	LN Metastasis	T size	Age	Treatment
PDA008	pT3N0	G2	29	A, NED	R0	0 of 18	4. 1cm	52	Gemcitabine, RTX
PDA046	pT3N1	G2	71	A, NED	R1	1 of 21	2.5 cm	69	None
PDA051	pT3N1	G2	31	A, NED	R1	4 of 15	3.0 cm	51	Chemotherapy
PDA081	pT3N1	G2	46	A, NED	R1	3 of 14	3.6 cm	63	Chemotherapy
PDA103	pT3N0	G2	17	A	R0	0 of 19	6.5 cm	84	None

Clinical pathological features of KRAS codon 61 mutated PDA cases: Representative hematoxylin and eosin staining of a Q61H (PDA_051) case. Clinical features of the codon 61 mutated cases with overall survival information. Scale bar is 100 µm.

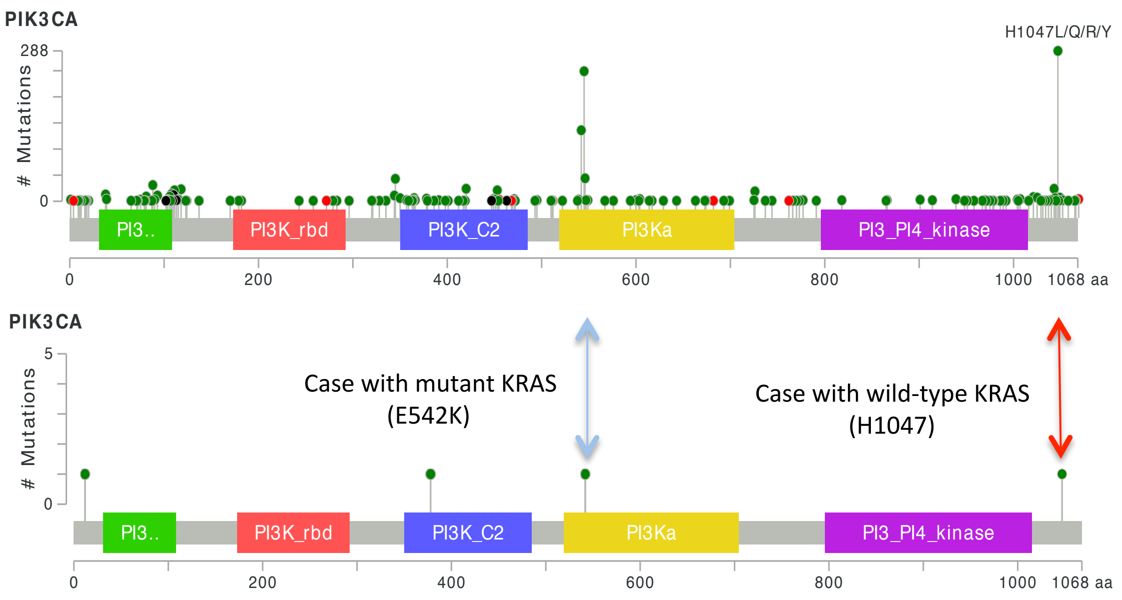

PEG Plots of PIK3CA in pancreatic cancer: Multiple distinct point mutation in PIK3CA were identified in the PDA cohort and compared relative to all cancer data in CBIOPORTAL. Two of the mutation occurred at known oncogenic hotspots for mutation in breast and other cancers (E542 and H1047). Only the mutation at H1074 occurred in a case with wild-type KRAS.

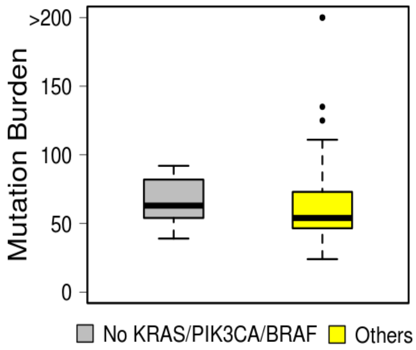

Case	Cancer Associated Mutation
PDA_049	CHEK2, TP53
PDA_043	GNAS, TCF4
PDA_077	STK11, AXIN1, CHEK2
PDA_080	RB1, STK11
PDA_068	NF1, SMO, SMAD4, GNAS

Cancer mutations present in KRAS/BRAF/PIK3CA wild-type tumors: 5 out of 109 cases did not have a detectable oncogenic variant at KRAS, BRAF, or PIK3CA. (Left) These cases exhibited similar number of mutations (SNV/INDEL) relative to other cases in the sequencing cohort. The boxes show the distance between the first and third quartile with the whiskers extending up to 1.5 times the interquartile range (Right Panel) These wild-type KRAS, BRAF, PIK3CA cases harbored mutations in a number of key cancer genes.

Supplementary Figure 29

Gene	*KRAS*	*PIK3CA*	*BRAF*
KRAS	---	0.2952	0.0004
PIK3CA		---	0.8929
BRAF			---

Nature 2012

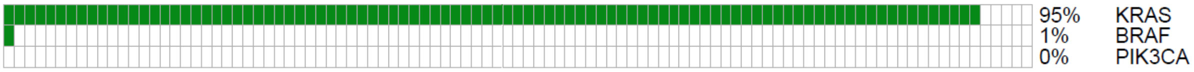

Mutual exclusivity assessment of BRAF and KRAS in pancreatic cancer. A Fisher exact test was used to determine mutual exclusivity between KRAS, BRAF, and PIK3CA mutations in the PDA cohort. Only BRAF was significant as being mutually exclusive (p-value determined by Fisher exact test).

Supplementary Figure 30

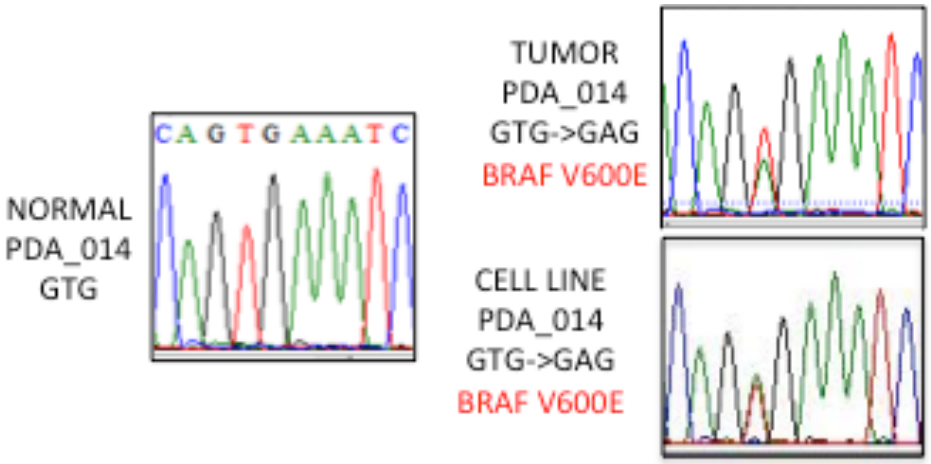

Validation of BRAF V600E-positive tumor cell line: A cell line was developed from the case PDA_014. This cell line harbored the V600E allele as did the primary tumor from which it was developed.

Supplementary Figure 31

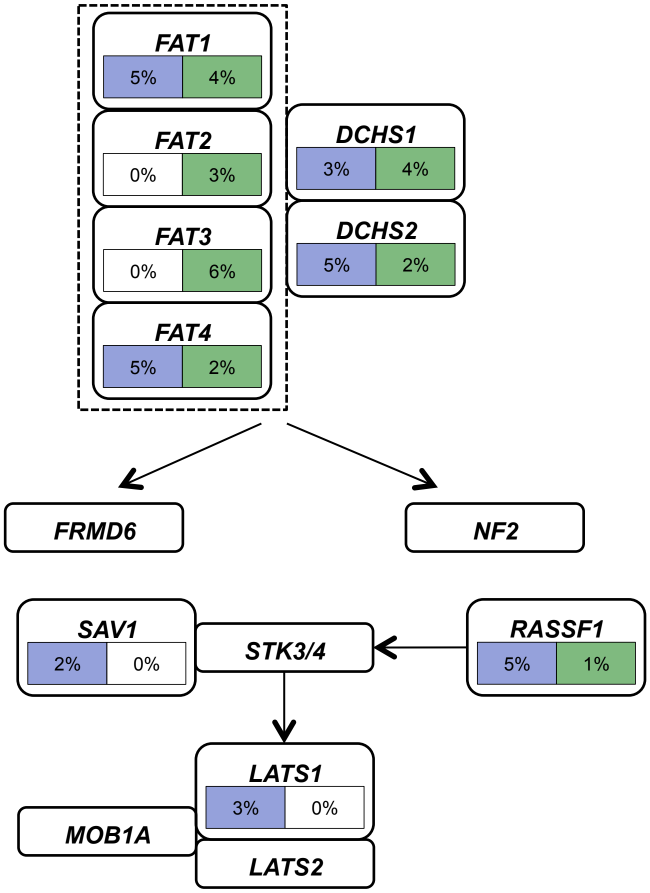

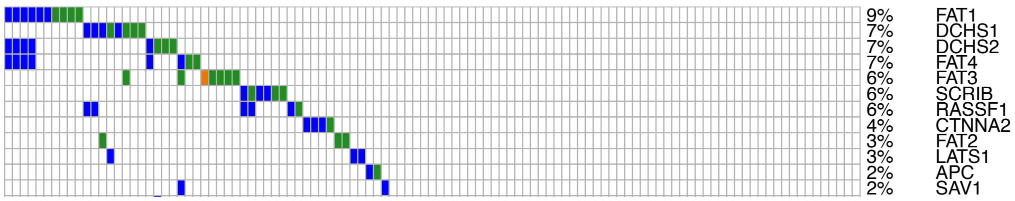

Mutations with the FAT/HIPPO pathway in pancreatic cancer: Diagram of the HIPPO pathway denoting mutations (green) and homozygous deletion (blue) for the genes indicated. Oncoprint showing the mutations in the FAT/HIPPO pathway.

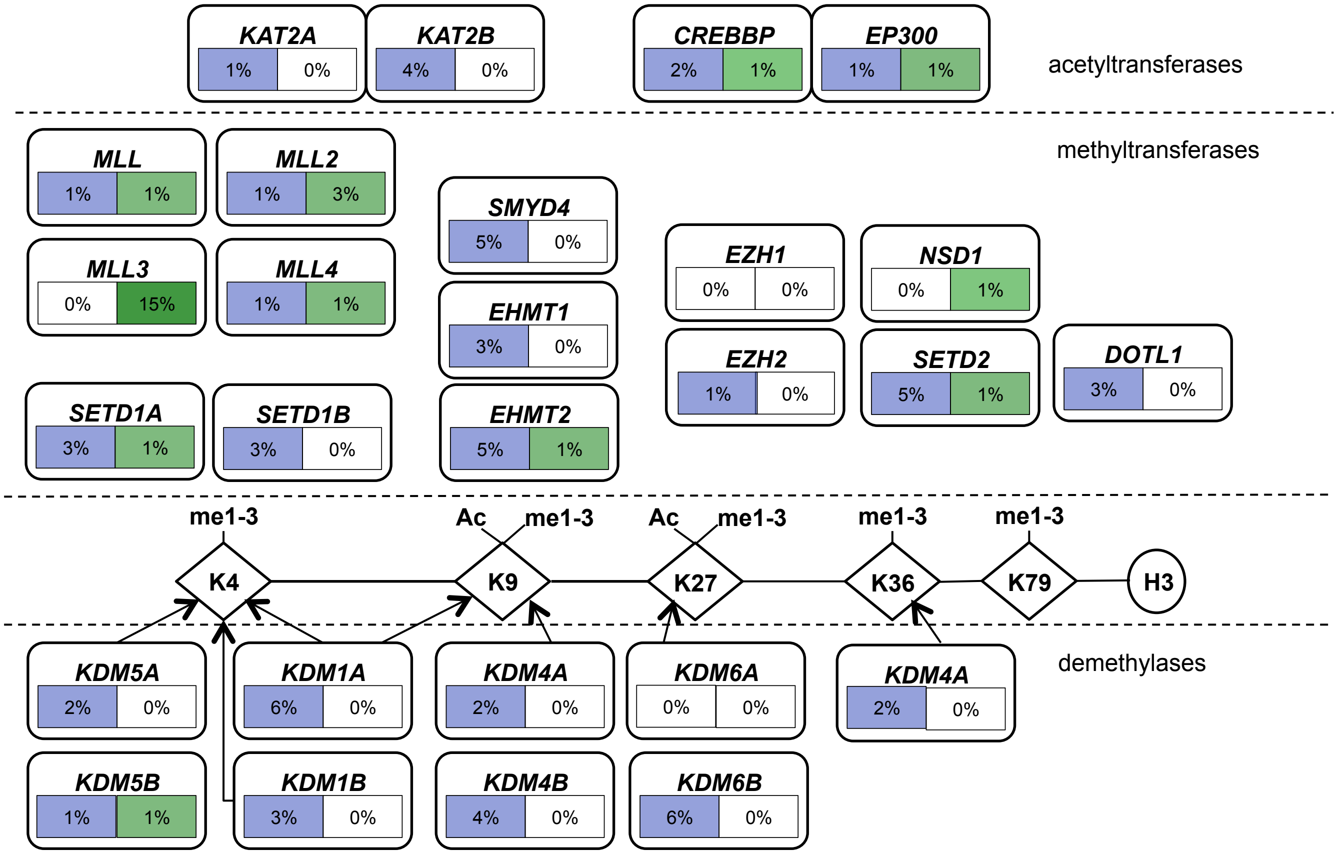

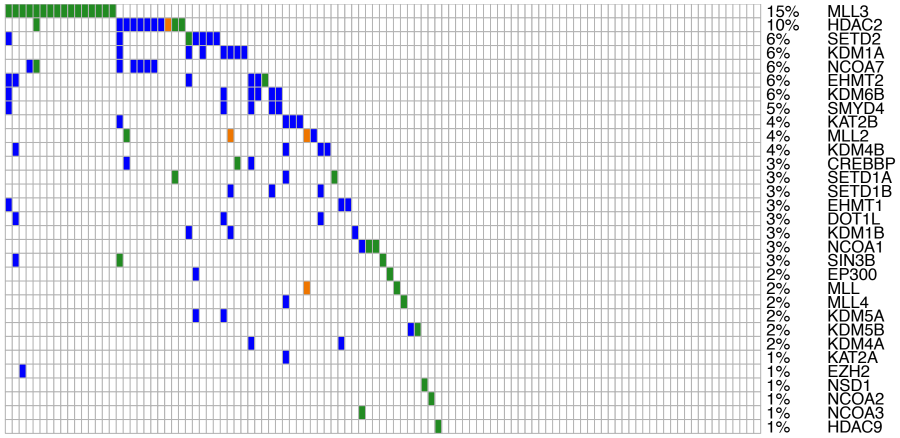

Mutations of histone modifying enzymes in pancreatic cancer: Diagram of histone modifying enzymes with mutations (green) and homozygous deletion (blue) for the genes indicated. Oncoprint showing the mutations in histone modifying enzymes.

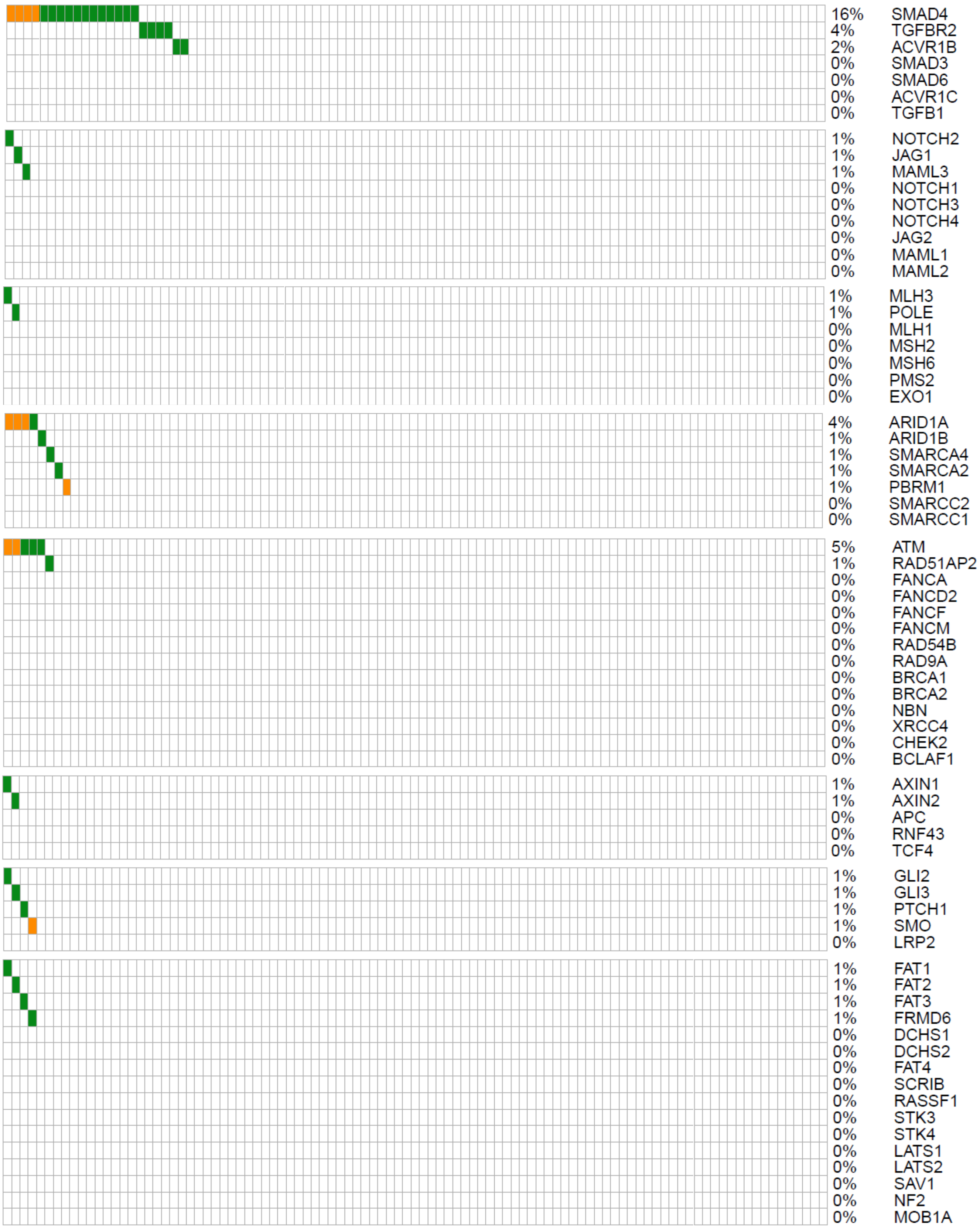

Oncoprints of pathways defined in the present study with data from Nature 2012: Oncoprints summarizing the pathways defined in the present study represented using data from the Nature 2012 study.

Supplementary Figure 34

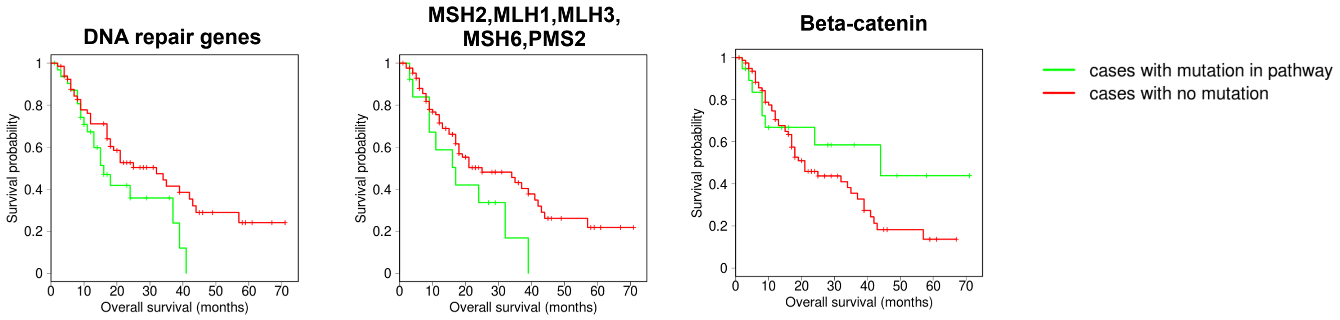

	# mutations	# no mutations	HR	Log Rank P-value
**Ras Pathway**	98	4	0.4788	0.1493
KRAS	94	8	0.5718	0.1921
**Mismatch Repair** (MSH2,POLE,MLH1,MLH3,EXO1,MSH6,PMS2)	20	82	1.4142	0.2725
MSH2,MLH1,MLH3,MSH6,PMS2	13	89	1.8347	0.0813
**TGFBeta Pathway**	57	45	1.0329	0.9046
SMAD4, TGFBR2	51	51	1.0067	0.98
SMAD4	44	58	0.8566	0.5626
**RB Pathway**	55	47	1.2135	0.4669
CDKN2A	43	59	1.0868	0.7663
**DNA damage Pathway (All)**	41	61	1.3761	0.2352
DNA repair genes vs. Others	34	68	1.709	0.0598
ATM/CHEK2 vs. Others	11	91	0.9219	0.8419
**SWI/SNF complex**	33	69	0.9389	0.8229
**HIPPO/FAT Pathway**	41	61	0.9195	0.7585
**Notch Pathway**	40	62	1.0581	0.8357
**Beta-catenin Pathway**	21	81	0.6309	0.2263
**Hedgehog Pathway**	23	79	1.15	0.6426

	**ASSOCIATION WITH:**					
	**Grade 3**		**Adenosquamous**		**Lymph Node Status**	
**Pathway**	**pvalue**	**OR**	**pvalue**	**OR**	**pvalue**	**OR**
RAS	0.3297		1		0.1161	4.4243
TGF-Beta	0.3794	1.5358	0.3389	2.3416	1	0.9931
HIPPO/FAT	0.6507	1.3127	0.1078	2.9821	1	1.09
MSI	0.2704	0.4473	0.1185	0	1	1.1981
DNA damage	0.6557	1.2474	1	0.83	1	0.9449
Beta-catenin	0.2762	1.8689	0.0519	3.6465	0.6079	0.7875
Cell cycle	0.0447	2.7247	0.5321	1.6722	0.3904	1.4986
Notch	0.5	1.3824	1	0.9031	1	1.0349
Hedgehog	1	1.0157	0.2543	2.2094	0.7957	0.8494
SNF complex	0.0902	2.3042	0.7319	1.3556	0.1586	0.5076
MYC	0.1063	2.6152	0.0005	12.8915	0.5173	0.6115
TP53	0.3742	1.6119	0.0619	4.27	0.8294	1.1394
Histone modification	0.1777	2.038	0.7573	1.3093	0.6638	0.7874

Statistical summary of the association of specific pathway alterations with survival in pancreatic cancer: The association of the indicated pathways features with overall survival, histological and pathological features of disease are shown (p-values and hazard ratio are by cox proportionality). Trends or significant associations are highlighted in blue (p-values and odds ratios were determined by Fisher's exact test).

Supplementary Figure 35

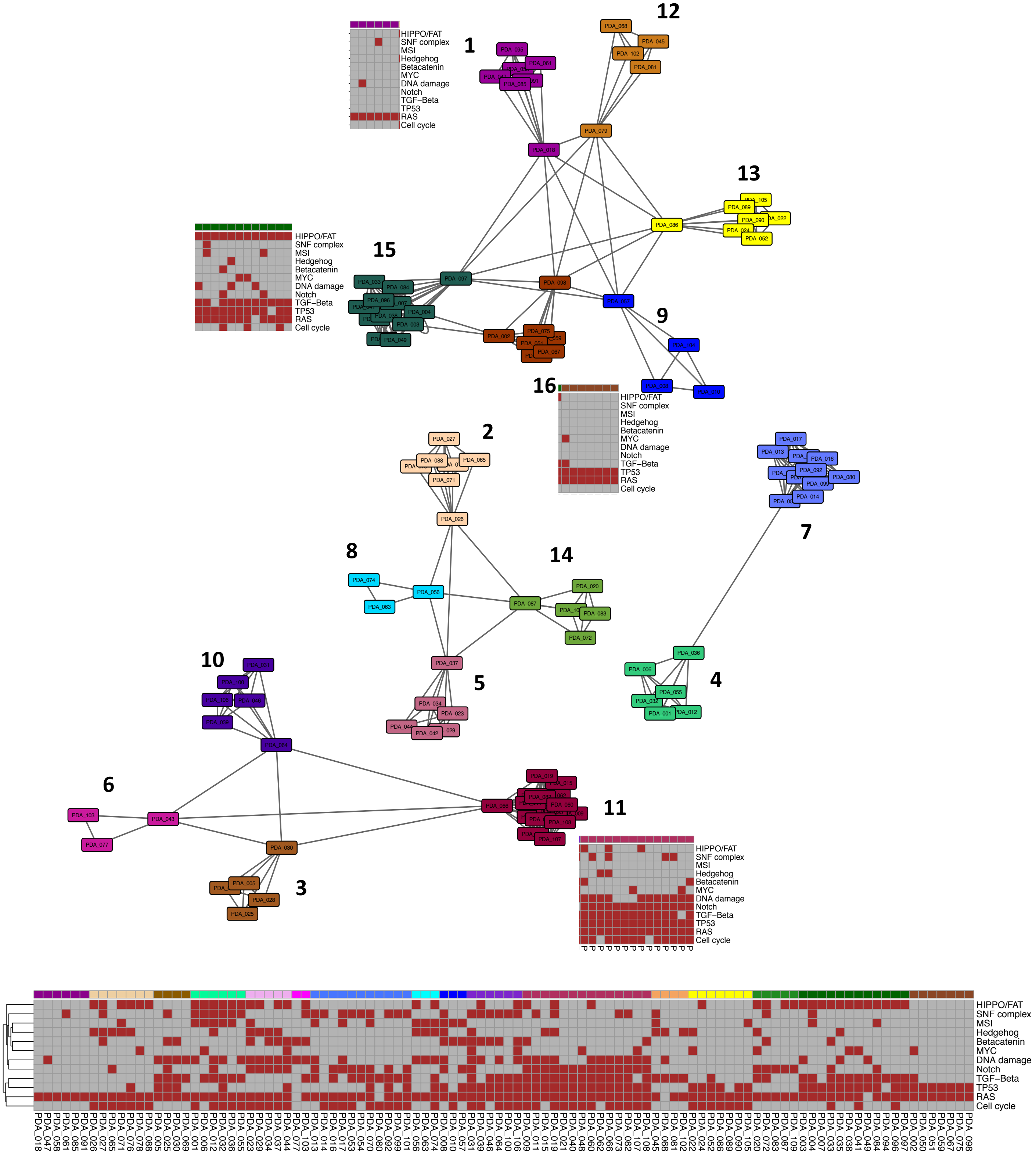

APC clustering of pathway alterations in pancreatic cancer: APC clustering generated networks as summarized by the heatmap. Select clusters are shown, including those dominated solely by RAS pathway alterations, as well as those with more complex genetic alterations.

Supplementary Figure 36

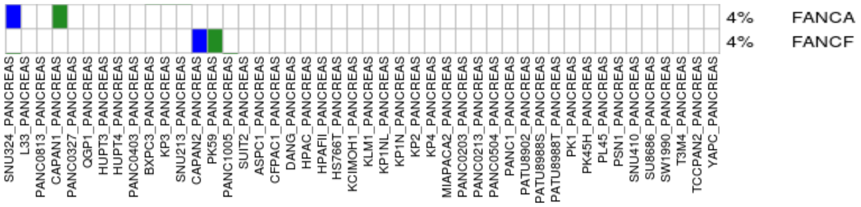

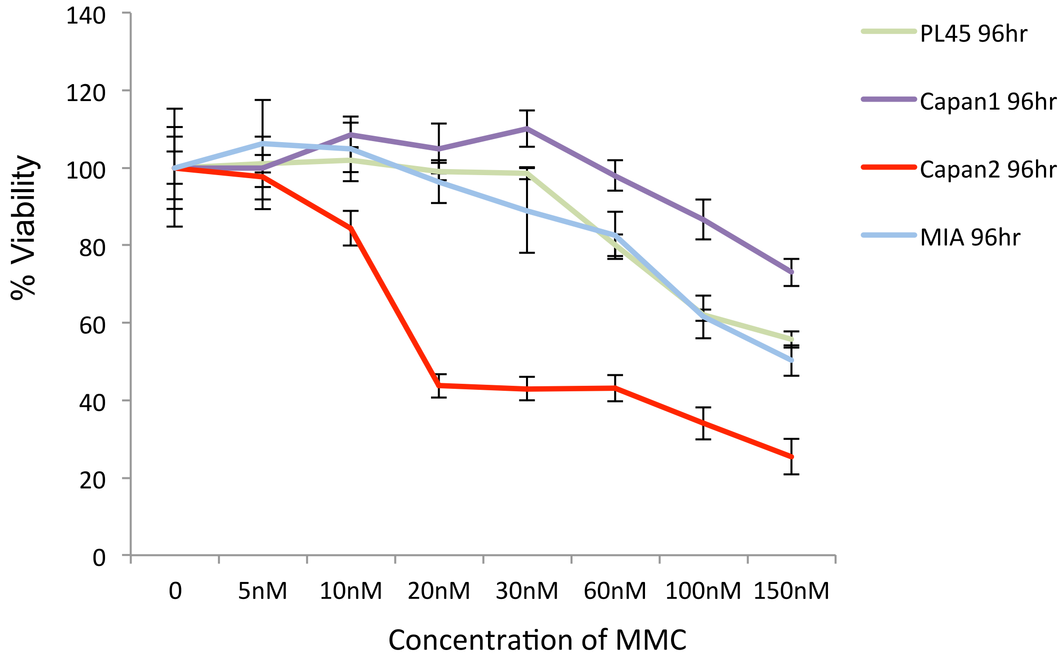

Selective sensitivity of cells harboring FANC mutations to mitomycin C: Select PDA cell lines exhibit deficiency in FA genes as was observed in sequencing of clinical cases. Cell lines with FANCF loss exhibit enhanced sensitivity to mitomycin C (IC50, ~20 nM) relative to cell lines with intact FANC complex (IC50, >150 nM). Error bars constitute the standard deviation in the values.

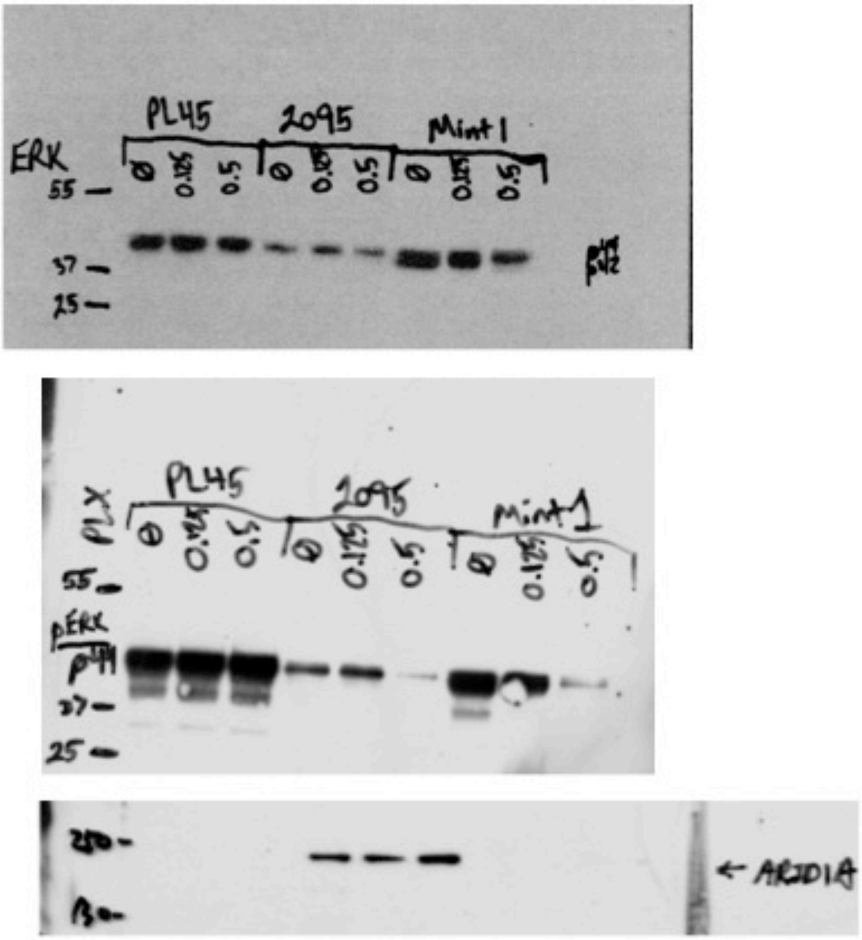

Uncropped relevant blots: total ERK, phospho-ERK, and Arid 1A.

Supplementary Table 1

Association with grade 3		
**Gene**	**pvalue**	**OR**
FLG	0.0046	6.6682
SMAD4	0.0931	0.2678
PIK3CA	0.2558	3.1585
GNAS	0.333	0
ARID1A	0.3613	2.4141
KRAS	0.4468	2.7901
RBM10	0.5702	0
IRF6	0.5702	0
HDAC2	0.5702	0
PABPC1	0.5702	0
CDH13	0.5702	0
AXIN1	0.5958	2.0901
CDKN2A	0.6362	1.553
RNF43	0.6362	1.553
RP1L1	0.6362	1.553
ITGAE	0.6362	1.553
SPTA1	0.6362	1.553
TP53	0.6582	1.3092
ATXN1	1	1.0132
COL14A1	1	1.2296
MLL3	1	1.2296
GLI3	1	0.5948
BCLAF1	1	0.7519
NIN	1	0.7519
USP10	1	1.0127

Association with adenosquamous		
**Gene**	**pvalue**	**OR**
FLG	0.0133	7.1917
TP53	0.2022	2.8669
PIK3CA	0.3507	3.1166
USP10	0.3507	3.1166
AXIN1	0.4187	2.3252
SMAD4	0.4403	1.6579
CDKN2A	0.4802	1.8468
RNF43	0.4802	1.8468
ITGAE	0.4802	1.8468
GLI3	0.4802	1.8468
ATXN1	0.5858	1.2966
KRAS	0.594	-
ARID1A	1	0
COL14A1	1	0
RP1L1	1	0
GNAS	1	0
SPTA1	1	0
BCLAF1	1	0
NIN	1	0
PARP14	1	0
RBM10	1	0
IRF6	1	0
HDAC2	1	0
PABPC1	1	0
CDH13	1	0

Association with nodal status		
**Gene**	**pvalue**	**OR**
GLI3	0.042	0.1636
SMAD4	0.1815	2.4978
GNAS	0.1902	0.3417
PIK3CA	0.2874	0.3502
IRF6	0.5717	-
HDAC2	0.5717	-
USP10	0.5717	-
CDH13	0.5717	-
AXIN1	0.6074	0.5294
NIN	0.6074	0.5294
RP1L1	0.6557	0.7129
ITGAE	0.6557	0.7129
SPTA1	0.6557	0.7129
COL14A1	0.6724	2.2562
ATXN1	0.6789	2.6657
KRAS	0.6975	1.4182
TP53	0.8307	1.1251
FLG	1	0.9633
ARID1A	1	0.9009
CDKN2A	1	1.8575
RNF43	1	1.8575
BCLAF1	1	1.469
PARP14	1	1.469
RBM10	1	1.0901
PABPC1	1	1.0901

Association of significantly mutated genes with pathological features: Each gene identified as significantly mutated by MutsigCV was evaluated for association with grade adenosquamous histology, and nodal status (p-values and odds ratios were determined by Fisher's exact test). Significant associations are highlighted.

Supplementary Table 2

**ARID1A-IHC Validation Cohort**	
**Characteristics**	No of patients 296 (%)
**Median Age (range)**	66 (38-89)
**Gender**	
**Male**	161 (54)
**Female**	135 (46)
**Tumor size (cm)**	
**0-2.0**	52 (17)
**2.1-4.0**	163 (55)
**> 4.0**	70 (24)
**Unknown**	11 (4)
**Node involvement**	
**Positive**	215 (73)
**Negative**	76 (26)
**Unknown**	5 (1)
**TNM Stage**	
**Ia**	12 (4)
**Ib**	19 (6)
**IIa**	35 (12)
**IIb**	171 (57)
**III**	23 (8)
**IV**	3 (1)
**Unknown**	6 (2)
**Vital Status**	
**Alive**	152 (51)
**Dead**	125 (42)
**Unknown**	19 (6)

Demographic table for ARID1A IHC cohort: A cohort of 296 cases was used to evaluate the association of ARID1A status with overall survival. The summary of clinicopathological characteristics is shown.
